# The Mevalonate Pathway: Innovations, Applications, and Challenges in Biotechnology with Emphasis on Fungal Biology

**DOI:** 10.3390/jof12070497

**Published:** 2026-07-07

**Authors:** Aisel Valle Garay, Cíntia Marques Coelho, Napoleão Fonseca Valadares, Leonardo Ferreira da Silva, Letícia Sousa Cabral, Matheus de Castro Leitão, Luiza Cesca Piva, Janice Lisboa De Marco, Brenda Rabello de Camargo, Amanda Araújo Souza, Izadora Cristina Moreira de Oliveira, Matheus Ferroni Schwartz, Túlio Marcos Godoy de Andrade, Talita Souza Carmo, João Ricardo Moreira de Almeida, Fernando Araripe Gonçalves Torres, Sonia Maria de Freitas

**Affiliations:** 1 Laboratory of Molecular Biophysics, Department of Cell Biology, Institute of Biological Sciences, University of Brasília (UnB), Asa Norte, Brasília-DF 70910-900, Brazil; napo@unb.br (N.F.V.); amandabernasol@gmail.com (A.A.S.); izadorabiomed@gmail.com (I.C.M.d.O.); matheus.schwartz@gmail.com (M.F.S.); nina@unb.br (S.M.d.F.); 2 Laboratory of Biocatalyst and Bioprocess Engineering, Department of Cell Biology, Institute of Biological Sciences, University of Brasília (UnB), Asa Norte, Brasília-DF 70910-900, Brazil; talitacarmo@unb.br; 3 Laboratory of Synthetic Biology, Center for Molecular Biotechnology (C-Biotech), University of Brasília Science and Technology Park (PCTec), Asa Norte, Brasília-DF 70910-900, Brazil; cintiacoelhom@unb.br (C.M.C.); leonardo.ferreira.unb@gmail.com (L.F.d.S.); leticiascabral5@gmail.com (L.S.C.); leitao.math@gmail.com (M.d.C.L.); 4 Department of Genetics and Morphology, Institute of Biological Science, University of Brasília (UnB), Asa Norte, Brasília-DF 70910-900, Brazil; 5 Microbial Genetics and Biotechnology Laboratory, EMBRAPA Agroenergy, Parque Estação Biológica (PqEB), Ave. W3 Norte (Final), s/n, Edifício da EMBRAPA Agroenergia, Brasília-DF 70770-901, Braziltulio.andrade@colaborador.embrapa.br (T.M.G.d.A.); joao.almeida@embrapa.br (J.R.M.d.A.); 6 Pharmacy Department, Faculty of Health, University of Brasília (UnB), Asa Norte, Brasília-DF 70910-900, Brazil; janicedemarco@unb.br; 7 Biochemistry Laboratory, Center for Molecular Biotechnology (C-Biotech), University of Brasília Science and Technology Park (PCTec), Asa Norte, Brasília-DF 70910-900, Brazil; 8 Yeast Molecular Genetics Laboratory, Center for Molecular Biotechnology (C-Biotech), University of Brasília Science and Technology Park (PCTec), Asa Norte, Brasília-DF 70910-900, Brazil; brendarc@gmail.com (B.R.d.C.); ftorres@unb.br (F.A.G.T.); 9 Graduate Program of Microbial Biology, Institute of Biological Sciences, University of Brasília, Asa Norte, Brasília-DF 70910-900, Brazil

**Keywords:** mevalonate, fungi, yeast, isoprenoids, mevalonate pathway, metabolic engineering, applications of isoprenoid, production of isoprenoids

## Abstract

The mevalonate (MVA) pathway is a central metabolic route responsible for the biosynthesis of isoprenoids with broad biological and biotechnological relevance. Due to its importance, the MVA pathway has attracted increasing interest in studies of enzymatic regulation, structural biology, metabolic engineering, and synthetic biology, particularly in fungi. This review provides a comprehensive overview of the MVA pathway, addressing its distribution across different domains of life, evolutionary aspects, and metabolic organization, with emphasis in fungi. Special attention is given to the biochemical and structural characterization of MVA-pathway enzymes, including catalytic mechanisms, structural features, and regulatory processes. The methylerythritol phosphate pathway is also presented as an alternative route for isoprenoid precursor biosynthesis and discussed in terms of its taxonomic distribution and metabolic significance. Recent advances in synthetic biology, enzyme regulation, and pathway engineering are highlighted, emphasizing their contributions to metabolic engineering and synthetic biology. Special emphasis is given to fungi, in which the MVA pathway plays a central role in ergosterol biosynthesis, protein prenylation, and secondary metabolite production. Advances in the engineering of fungal cells, including *Saccharomyces cerevisiae* and other emerging fungal species, are discussed in the context of sustainable isoprenoid production. Finally, strategies for optimizing microbial production are presented, highlighting the importance of fungal synthetic biology in advancing biotechnological applications.

## 1. Overview of the Mevalonate Pathway: Distribution, Evolution, and General Organization

The mevalonate (MVA) pathway is a highly conserved metabolic route responsible for the biosynthesis of the universal isoprenoid precursors isopentenyl diphosphate (IPP) and dimethylallyl diphosphate (DMAPP). These five-carbon (C_5_) isoprene units are derived from the condensation of three molecules of acetyl-coenzyme A (acetyl-CoA) and are fundamental molecular building units for a wide and structurally diverse family of isoprenoids, encompassing between 30,000 and 80,000 known compounds distributed across all domains of life [1,2]. These include essential biomolecules such as terpenoids, polyisoprenoids (e.g., dolichols), sterols (e.g., cholesterol, lanosterol, ergosterol), ubiquinone, and several other metabolites required for cellular structure and function [3,4].

The MVA pathway is recognized as the predominant route for isoprenoid biosynthesis in eukaryotes, including animals, fungi, and the cytosol of plant cells, as well as in archaea and a limited number of eubacteria [5,6,7,8]. It establishes a critical metabolic link between central carbon metabolism and isoprenoid biosynthesis, bridging acetyl-CoA, a key product of glycolysis and other catabolic processes, to a wide range of downstream isoprenoid derivatives. Although historically considered the sole pathway for isoprenoid production, it is now known that alternative pathways, such as the methylerythritol phosphate (MEP) pathway, also contribute to isoprenoid biosynthesis in many organisms [1,2]. Across organisms harboring this pathway, acetyl-CoA acts as the primary carbon feedstock. Notably, acetate can be readily transported across cellular membranes and is often used biochemically as a precursor to fuel the pathway, in contrast to downstream intermediates, which are typically membrane-impermeable [4].

**Figure 1:**
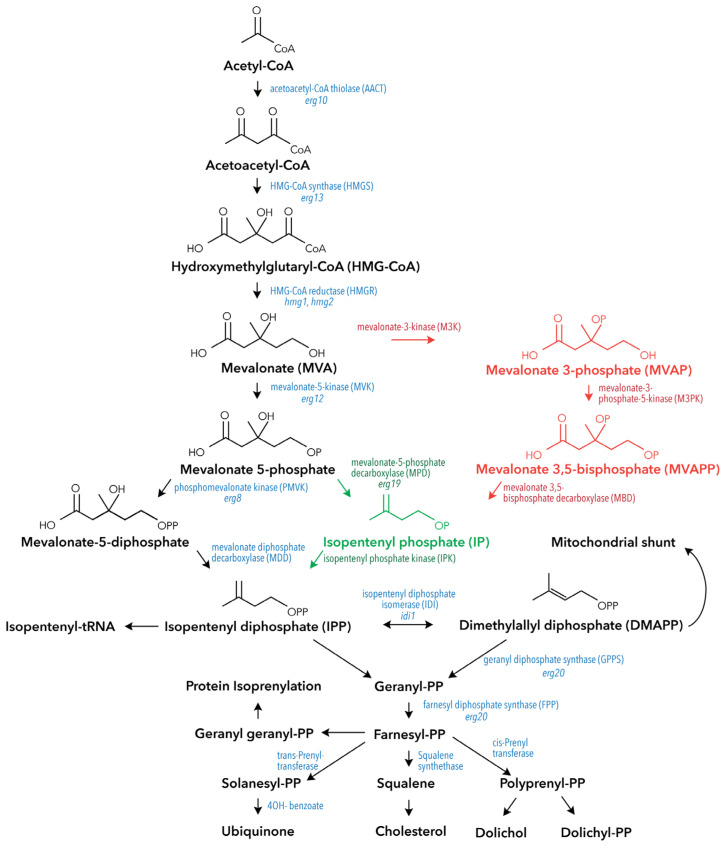
Overview of the mevalonate (MVA) pathway and its alternative branches. The canonical MVA pathway begins with the condensation of acetyl-CoA to form acetoacetyl-CoA, followed by conversion to 3-hydroxy-3-methylglutaryl-CoA (HMG-CoA) and its subsequent reduction to mevalonate by HMG-CoA reductase (HMGR). Mevalonate is then phosphorylated by mevalonate kinase (MVK) to yield mevalonate-5-phosphate, which is further converted to mevalonate-5-diphosphate and decarboxylated to produce isopentenyl diphosphate (IPP), the universal isoprenoid precursor. IPP is isomerized to dimethylallyl diphosphate (DMAPP), enabling the synthesis of downstream isoprenoids such as geranyl diphosphate (GPP), farnesyl diphosphate (FPP), and higher-order compounds including squalene, cholesterol, ubiquinone, and dolichols. In addition to the classical route (black/blue), alternative branches are highlighted: (i) archaea I pathway, a decarboxylation route generating isopentenyl phosphate (IP), later converted to IPP (green), and (ii) archaea II pathway, phosphorylation pathway via mevalonate-3-phosphate and mevalonate-3,5-bisphosphate (red). Enzymes and corresponding gene (in italic) annotations are indicated where applicable. This scheme emphasizes the metabolic flexibility and regulatory complexity of isoprenoid biosynthesis through the MVA pathway.

The MVA pathway comprises seven enzymatic steps that can be functionally divided into two major segments (Figure 1). The upper pathway converts acetyl-CoA into mevalonate, while the lower pathway converts mevalonate into the activated isoprene units IPP and DMAPP. In the upper segment, two molecules of acetyl-CoA are initially condensed by acetoacetyl-CoA thiolase to form acetoacetyl-coenzyme A (acetoacetyl-CoA). A third acetyl-CoA is subsequently incorporated by HMG-CoA synthase, yielding 3-hydroxy-3-methylglutaryl-coenzyme A (HMG-CoA) (Figure 1). This intermediate is then reduced to MVA in a key rate-limiting step catalyzed by HMG-CoA reductase, an enzyme subject to complex regulatory mechanisms and a major target of pharmacological inhibition [3,4].

In the lower segment of the pathway, mevalonate undergoes sequential phosphorylation reactions catalyzed by mevalonate kinase and phosphomevalonate kinase, followed by an ATP-dependent decarboxylation step mediated by mevalonate diphosphate decarboxylase (Figure 1). These reactions culminate in the formation of isopentenyl diphosphate (IPP), the central precursor of isoprenoid biosynthesis. IPP can then be isomerized by isopentenyl diphosphate isomerase to yield DMAPP, enabling the generation of diverse isoprenoid chains through successive condensation reactions. Additionally, IPP plays a role beyond classical isoprenoid biosynthesis, contributing to tRNA modification processes (Figure 1).

IPP and DMAPP serve as substrates for prenyltransferases that generate longer-chain isoprenoids such as geranyl pyrophosphate (GPP), farnesyl pyrophosphate (FPP), and geranylgeranyl pyrophosphate (GGPP) (Figure 1). These intermediates represent key metabolic branch points. For instance, FPP can be converted into squalene via squalene synthase, initiating the sterol biosynthesis pathway, which leads to the production of ergosterol in fungi, cholesterol and steroid hormones in animals, and phytosterols in plants [9]. Alternatively, FPP can be directed toward non-sterol isoprenoid biosynthesis, giving rise to a wide array of compounds such as dolichols, involved in protein glycosylation, and heme A, a critical component of the respiratory chain [10]. GGPP, in turn, participates in protein prenylation and the synthesis of diterpenoids and carotenoids, further expanding the functional diversity of isoprenoid products (Figure 1) [8]. Therefore, the MVA pathway serves as a central metabolic hub that integrates primary metabolism with the biosynthesis of structurally and functionally diverse isoprenoids, highlighting its biochemical versatility and fundamental importance in cellular physiology. Next, we present its detailed biochemical architecture, focusing on the individual enzymatic steps, catalytic mechanisms, structural features, and regulatory strategies that control its function.

## 2. Biochemical Architecture of the Mevalonate Pathway: Enzymatic Steps, Mechanisms, Structural Insights, and Regulation

### 2.1. The Upper Mevalonate Pathway: From Acetyl-CoA to Mevalonate

Step 1: Condensation of two acetyl-CoA by Acetyl-CoA Acetyltransferase.

The MVA pathway begins with the condensation of three acetyl-CoA molecules to form 3-hydroxy-3-methylglutaryl-CoA (HMG-CoA). The first reaction involves the reversible, non-decarboxylative condensation of two molecules of acetyl-CoA to form acetoacetyl-CoA and CoA-SH. This step is catalyzed by acetyl-CoA acetyltransferase (AACT; with enzyme commission number EC 2.3.1.9), also known as acetoacetyl-CoA thiolase, encoded by the *erg10* gene in many organisms (Figure 1) [11,12]. In mammals, the cytosolic AACT isoform participates in sterol and isoprenoid biosynthesis and is distinct from the mitochondrial thiolase isoform involved in ketone body metabolism. Fungi AACT enzymes show greater diversity: *Saccharomyces cerevisiae* has just one dedicated thiolase gene (*erg10*), but other fungi, like the filamentous *Aspergillus oryzae*, encodes a family of up to six thiolase genes [13]. The regulation of cytosolic AACT occurs primarily at the transcriptional level. Its expression is coordinated with other cholesterogenic enzymes via the sterol regulatory element-binding protein (SREBP) pathway, ensuring synchronized flux through the pathway in response to cellular sterol levels [14,15].

**Figure 2:**
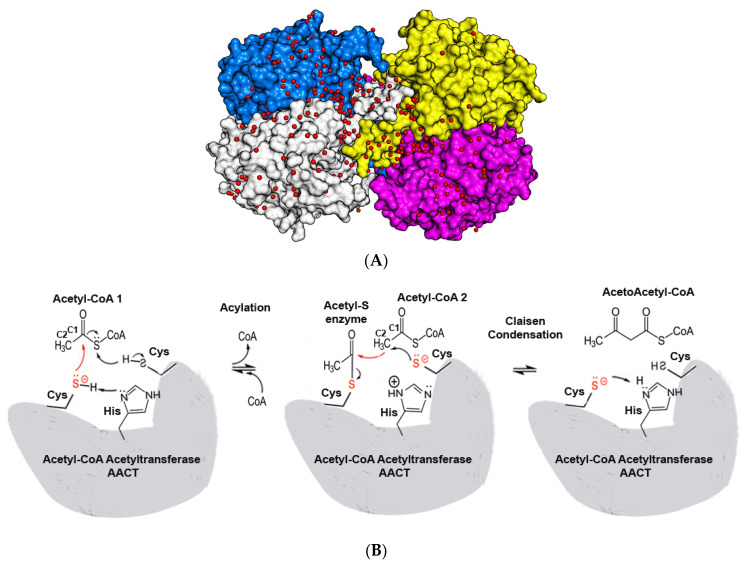
Structure and mechanism of acetyl-CoA acetyltransferase (AACT, acetoacetyl-CoA thiolase). The enzyme catalyzes the initial condensation of the mevalonate pathway. (**A**) The tetrameric acetyl-CoA acetyltransferase from *Saccharomyces cerevisiae*. The monomers are represented as molecular surfaces colored in blue, white, yellow and magenta (PDB: 5XZ5). Red spheres indicate water molecules. (**B**) The two-step catalytic mechanism by which AACT condenses two molecules of acetyl-CoA to form acetoacetyl-CoA. In the first step, the acetyl group from acetyl-CoA is transferred to the active site cysteine residue, generating a covalent acetyl-enzyme (acetyl-Cys) intermediate. This acylation step is facilitated by a conserved histidine residue acting as a general base. In the second step, the acetyl group bound to the cysteine undergoes a Claisen condensation reaction with a second molecule of acetyl-CoA, resulting in the formation of acetoacetyl-CoA and regeneration of the free enzyme. The shaded area represents the enzyme active-site, and key catalytic residues are highlighted. Black curved arrows indicate electron flow and H^+^ transference, and red nucleophilic attack within the active site.

To date, approximately 84 crystallographic structures of AACT have been deposited in the Protein Data Bank (PDB, https://www.rcsb.org/; search up to 14 April 2026), in apoenzyme forms, substrate- or inhibitor-bound complexes, and engineered variants. These structures span all domains of life (61 bacteria, 21 eukaryotic and two archaea). High-resolution structures (≤2.0 Å) are predominantly bacterial (27), but include important eukaryotic examples such as *Homo sapiens* (four). Particularly, AACT structures from *Zoogloea ramigera* (11) have been extensively studied because they support bacterial production of polyhydroxybutyrate. Collectively, the AACT structures illustrate a high degree of structural conservation and reveal multiple snapshots along the catalytic cycle. The AACT enzymes adopt a conserved tetrameric assembly, as observed in both fungal and human ERG10 homologs (Figure 2A) [12].

Catalysis proceeds through two chemically distinct steps following a classic Ping-Pong (double-displacement) mechanism, in which a nucleophilic cysteine residue forms a covalent acetyl-enzyme intermediate (Figure 2B). In the first step (acylation), the active-site cysteine attacks the C_1_ carbonyl carbon of the first acetyl-CoA molecule, leading to the formation of a stable thioester-linked acetyl-enzyme intermediate (acetyl-S-enzyme) and the release of CoA-SH. This covalent intermediate is a hallmark of thiolase-mediated reactions and primes the enzyme for carbon–carbon bond formation. In the second step (condensation), a Claisen-type condensation occurs. A second acetyl-CoA molecule is activated through deprotonation at the C_2_ methyl group by a second conserved cysteine residue, generating a reactive enolate intermediate. This nucleophile subsequently attacks the carbonyl carbon of the acetyl-enzyme intermediate, resulting in the formation of acetoacetyl-CoA and regeneration of the free enzyme (Figure 2B) [16].

This catalytic process is supported by a conserved Cys-Asn-His triad within the active site [17]. The histidine residue plays a critical role in stabilizing the oxyanion that develops on the thioester carbonyl during catalysis, thereby lowering the activation energy of the reaction. This strategy is commonly observed in enzymes catalyzing Claisen-type condensations involving acyl-CoA thioesters [4]. The Cys-Asn-His arrangement is characteristic of thiolase-family enzymes and resembles catalytic motifs found in other “initial condensation enzymes”. Notably, it can be compared to the Cys-His-Asn triad described for the β-ketoacyl-ACP synthase involved in bacterial type II fatty acid biosynthesis [18]. A related variation in this catalytic architecture is also observed in HMG-CoA synthase, which catalyzes the subsequent step in the mevalonate pathway.

Step 2: Condensation of acetoacetyl-CoA and acetyl-CoA by HMG-CoA synthase.

**Figure 3:**
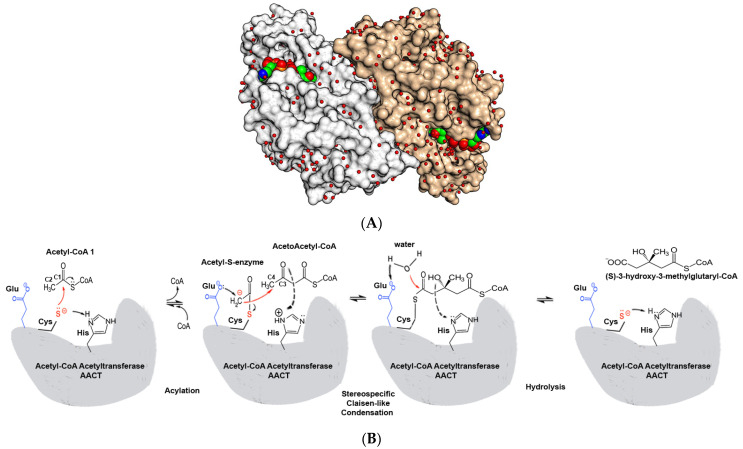
Structure and mechanism of HMG-CoA synthase. (**A**) Structure of HMG-CoA synthase from *Homo sapiens* in complex with CoA-SH. The homodimer is represented as molecular surfaces with beige and white subunits. CoA-SH is depicted in green. Red spheres indicate water molecules (PDB: 2P8U). (**B**) Catalytic mechanism of HMG-CoA synthase in the mevalonate pathway. Schematic representation of the stepwise reaction mechanism catalyzed by HMG-CoA synthase, showing condensation of acetyl-CoA with acetoacetyl-CoA to form 3-hydroxy-3-methylglutaryl-CoA (HMG-CoA). The mechanism proceeds through three main stages: (1) formation of an acetyl-enzyme thioester intermediate involving the catalytic cysteine residue (Cys), with release of CoA-SH; (2) nucleophilic condensation with acetoacetyl-CoA to generate a covalent intermediate; and (3) hydrolysis and product formation, yielding HMG-CoA. Key active-site residues (Cys, His, and Glu (in blue)) are highlighted, illustrating their roles in proton transfer, stabilization of intermediates, and catalysis. Black curved arrows indicate electron flow and H^+^ transference, black dashed line hydrogen bond, and red nucleophilic attack within the active site.

The second condensation reaction converts acetoacetyl-CoA and a third acetyl-CoA into 3-hydroxy-3-methylglutaryl-CoA (HMG-CoA). This reaction is catalyzed by HMG-CoA synthase (HMGS; EC 2.3.3.10), encoded by the *erg13* gene in *S. cerevisiae* (Figure 1). In bacteria, the enzyme is often annotated as mevalonate pathway enzyme S (*mvaS*), *hmgS*, or HMGS, and it is also present in the cytosol of plants. In eukaryotes, particularly mammals, HMGS exists in two isoforms: a cytosolic enzyme (HMGCS1) that supplies HMG-CoA for the mevalonate pathway, and a mitochondrial isoform (HMGCS2) that participates in ketogenesis [19]. Mitochondrial HMGCS2 is regulated by peroxisome proliferator response elements (PPREs) and plays a central role in hepatic ketogenesis [19,20]. Both isoforms of the human enzyme have been solved by X-ray crystallography, revealing a dimeric assembly (Figure 3A).

To date, 27 HMGS crystal structures are available in the PDB (search up to 14 April 2026), derived from bacteria (18), eukaryotes (seven) and archaea (two). The 12 high-resolution structures (≤2.0 Å) are predominantly derived from bacteria (nine), particularly from *Enterococcus faecalis* (three), and *Homo sapiens* (one). These structures mainly reveal a conserved homodimeric architecture (Figure 3A), with active sites located at the monomer–monomer interface. Although bacterial and mammalian HMGS structures are well characterized, no fungal HMGS structure is currently available.

HMGS catalyzes a stereospecific Claisen-like condensation of acetyl-CoA and acetoacetyl-CoA that yields (S)-HMG-CoA [4,21]. The reaction follows a Ping-Pong mechanism involving a conserved Cys-His-Glu catalytic triad and a covalent acetyl-enzyme intermediate [19,22,23]. The first step begins with the binding of acetyl-CoA to the active site. The thiol group of a conserved catalytic cysteine residue, ionized by His, performs a nucleophilic attack on the thioester carbonyl (carbon C_1_) of acetyl-CoA. This results in the formation of a covalent thioester bond between the acetyl group and the enzyme (acetyl-enzyme intermediate) and the release of CoA-SH (Figure 3B). This step effectively “primes” the enzyme with an activated acetyl group.

In the second step, acetoacetyl-CoA binds in proximity to the acetylated cysteine. A general base in the active site (often a conserved glutamate residue) abstracts a proton from the α-carbon (C_2_) of acetyl group in the acetyl-enzyme thioester, generating a resonance-stabilized enolate intermediate (Figure 3B). This enolate is the key nucleophile for subsequent C-C bond formation and is stabilized by oxyanion hole-like interactions in a tunnel-like active site with precise positioning of both CoA substrates. The activated enolate of the acetyl-enzyme thioester performs a nucleophilic attack on the carbonyl carbon (C_3_) of acetoacetyl-CoA (Figure 3B). This step forms a new carbon–carbon bond (Claisen condensation), generating a tetrahedral intermediate bound to the enzyme.

The stereochemical course of this step is tightly controlled by the active-site geometry, which orients the enolate and electrophile in a defined spatial arrangement. Finally, the tetrahedral intermediate collapses, leading to hydrolysis of the thioester bond by a water molecule, releasing the active enzyme and forming HMG-CoA (Figure 3B) [22,23,24,25]. The enzyme enforces strict stereochemical control, yielding exclusively the (S)-enantiomer of HMG-CoA (Figure 3B) [4,23,25].

Step 3: Reduction in HMG-CoA by HMG-CoA Reductase (HMGR).

The third reaction of the MVA pathway is a stereospecific enzymatic reduction in the thioesterified (S)-HMG-CoA carboxyl group to an alcohol, the (R)-mevalonate. This three-stage reaction involves two NADPH-dependent reductions catalyzed by HMG-CoA reductase (3-hydroxy-3-methylglutaryl-CoA reductase) (HMGR, EC 1.1.1.34, *hmgR* gene) (Figure 1) in eukaryotes, archaebacteria, and some eubacteria [4]. In bacteria and archaea, the genes are also commonly annotated as mevalonate pathway enzyme A (mvaA). This enzyme belongs to the dehydrogenase/reductase family and represents the rate-limiting and most tightly regulated step of the pathway. In mammals, HMGR is a membrane-bound enzyme of the endoplasmic reticulum encoded by a single gene with strong regulatory control by sterol feedback via the sterol regulatory element-binding protein (SREBP). Yeast possesses two HMGR isoforms (HMGR1 and HMGR2), derived from gene duplication, with distinct regulatory properties and stability [4,15,26].

**Figure 4:**
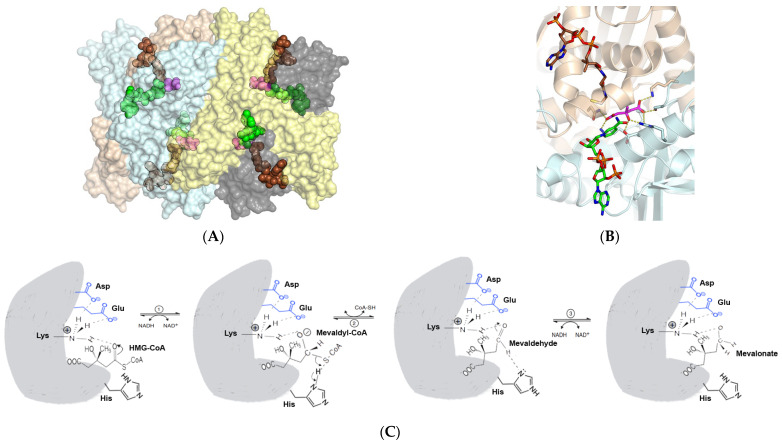
Structure and mechanism of HMG-CoA reductase. Crystal structure of HMG-CoA reductase from *Homo sapiens* in complex with CoA, HMG and NADP^+^. (**A**) Overview of the tetrameric enzyme shown the protein subunits as transparent molecular surfaces colored in light cyan, beige, pale yellow, and light gray, respectively. The four active sites include CoA molecules colored brown, HMG shown in magenta, and NADP+ depicted in green. (**B**) Close-up view of one active site formed at the interface between two subunits (beige and cyan), represented in cartoon. The ligands CoA (brown), HMG (magenta) and NADP^+^ (green) are shown, with key residues involved in HMG binding represented as sticks. PDB entry 1DQA. (**C**) Proposed reaction mechanism for HMGR. The side chains of the key catalytic residues, Lys, Asp, Glu, and His, are labeled, and the substrate and products are shown. The reaction follows three stages. Black curved arrows indicate electron flow and black dashed line hydrogen bond.

More than 30 HMGR crystal structures have been deposited in the PDB (search up to 14 April 2026) from bacteria (four), eukaryotes (28) and archaea (three). The three dimensional high-resolution crystal structures (≤2.0 Å) are derived from eukaryote *Homo sapiens* (two) and *Arabidopsis thaliana* (one) and exhibit homotetrameric structures (Figure 4A). The HMGRs of different organisms are multimers of a species specific number of identical monomers, mainly homodimers with residues from two different subunits that contribute to an active site formed at the interface [4] (Figure 4B).

HMGRs are classified into two major classes according to their structural and phylogenetic characteristics. Class I HMGRs are mainly distributed among eukaryotes and archaea, whereas Class II HMGRs are predominantly found in bacteria. In mammals and many other eukaryotic organisms, Class I HMGRs are membrane-associated proteins anchored to the endoplasmic reticulum and composed of an N-terminal transmembrane regulatory domain containing a sterol-sensing domain (SSD), as well as a highly conserved C-terminal soluble catalytic domain. This catalytic region is the primary target of statins, a class of drugs widely used to inhibit sterol biosynthesis [4]. In addition to their endoplasmic reticulum localization, HMGR enzymes have also been detected in rat liver peroxisomes [27,28]. In contrast, soluble Class I HMGR forms lacking membrane-associated regions have been reported in certain archaeal and fungal species [26,29,30].

Despite low sequence identity in HMGRs (<20%), the three-dimensional fold and catalytic residues are highly conserved. They have an NADPH-binding fold known as a non-Rossmann-type coenzyme-binding motif, a structural fold that binds nicotinamide dinucleotides and is found in many enzymes that use NADH and NADPH for catalysis [4]. The active site of HMG-CoA reductase is at the interface of the homodimer between one monomer that binds the NADPH and a second monomer that binds the HMG-CoA [26]. Different residues conserved in both classes of HMGRs, such as Lys, Asp, Glu, and His, form a conserved catalytic network between the two subunits that binds (S)-HMG-CoA, stabilizes reaction intermediates, orients substrates, and mediates proton transfer reactions [4]. Although the crystal structure of the yeast catalytic domain has not been determined, the landmark structures of human HMGR, solved in complex with HMG, NADP^+^, and CoA-SH (Figure 4B), and subsequently with six clinically approved statins, provide the primary structural framework for the fungal enzyme [29].

The human HMGR uses NADPH as a coenzyme and crystallizes as a homotetramer. The catalytic Lys is found on the monomer that binds the HMG-CoA and contributed by the so-called cis-loop (a section that connects the HMG-CoA-binding region with the NADPH-binding region). In contrast, the *Pseudomonas mevalonii* HMGR uses NADH as a coenzyme, lacks the cis-loop, and the catalytic Lys is present in the monomer that binds NADH. This enzyme crystallizes as a trimer of dimers [26].

HMGR catalysis proceeds through two sequential hydride-transfer reactions that use NADPH (Class I, eukaryotic HMGR) or NADH (Class II, prokaryotic HMGR) as a cofactor, converting (S)-HMG-CoA into (R)-mevalonate via a thiohemiacetal intermediate (mevaldyl-CoA) and a transient aldehyde intermediate (mevaldehyde) (Figure 4C). Upon substrate binding, (S)-HMG-CoA is positioned within the active site such that its thioester carbonyl is polarized by a conserved lysine residue. This electrostatic interaction enhances the electrophilicity of carbonyl carbon, facilitating hydride attack from NAD(P)H. A network of acidic residues, typically Asp and Glu, forms hydrogen bonds with Lys, contributing to precise substrate orientation and stabilization of catalytic intermediates. In parallel, a conserved histidine residue is strategically positioned to mediate proton transfer events throughout the reaction (Figure 4C).

In the first reduction step, a hydride from NAD(P)H is transferred to the thioester carbonyl carbon of HMG-CoA, yielding the thiohemiacetal intermediate (mevaldyl-CoA). During this process, the lysine residue stabilizes the developing oxyanion, while Asp and Glu maintain proper positioning and proton relay. The histidine residue likely contributes by facilitating protonation of the alkoxide intermediate, thereby stabilizing the thiohemiacetal [4,26,29]. Subsequently, the thiohemiacetal intermediate collapses, leading to cleavage of the thioester bond, release of CoA-SH, and formation of the aldehyde intermediate, mevaldehyde. This step involves protonation of the departing thiolate, a process mediated by the catalytic network in which histidine acts as a proton donor and lysine stabilizes the transition state (Figure 4C). In the final step, a second molecule of NAD(P)H binds to the enzyme and donates a hydride to the aldehyde carbon of mevaldehyde, producing (R)-mevalonate. As in the first reduction, lysine stabilizes the oxyanion intermediate, while Asp and Glu preserve the hydrogen-bonding architecture required for efficient catalysis. Histidine again participates in proton transfer to ensure completion of the reduction [4,26,29] (Figure 4C).

Overall, HMGR catalysis relies on a finely coordinated interplay among key active-site residues: Lys functions in electrostatic polarization and transition-state stabilization, Asp and Glu maintain substrate positioning and hydrogen-bonding networks, and His mediates proton transfer. The reaction requires two equivalents of NAD(P)H, proceeds through covalent thioester-derived intermediates within a closed enzyme conformation, and exemplifies an efficient enzymatic strategy for stepwise reduction in thioesters to alcohols [4,26,29].

Metabolic Regulation on HMGRs

The HMGR is the key rate-limiting enzyme of the MVA pathway and plays a central role in controlling the cellular levels of sterols and non-sterol isoprenoids. HMGR activity is tightly regulated at multiple levels, including transcription, translation, post-translational modification, and protein degradation, ensuring precise control of mevalonate-derived metabolites. At the transcriptional level in eukaryotes, HMGR expression is primarily controlled by sterol regulatory element-binding proteins (SREBPs), which are activated under sterol-depleted conditions and upregulate not only HMGR but also a broad set of genes involved in cholesterol and isoprenoid biosynthesis. In addition, the rate of HMGR translation is influenced by cellular demand for non-sterol isoprenoids, while protein stability is modulated by sterol availability, linking enzyme abundance directly to metabolic needs [3,26,29,31,32,33]. Furthermore, the reaction catalyzed by human HMG-CoA reductase is a key target for anti-hypercholesterolemic drugs (statins), which are structural analogs of the reaction intermediate and reduce cardiovascular risk by lowering serum cholesterol [26,29].

Structurally, eukaryotic HMGR is an integral endoplasmic reticulum (ER) membrane protein containing seven transmembrane helices in its N-terminal domain, with the catalytic C-terminal domain projecting into the cytosol [34,35]. The N-terminal region participates in sterol sensing and regulatory interactions, including glycosylation of luminal loops; whereas, the highly conserved cytosolic domain carries out catalysis. Enzyme activity can also be modulated by reversible phosphorylation, likely at serine residues near the C-terminus, providing a rapid mechanism to adjust catalytic efficiency in response to cellular signals [28].

In fungi, HMGR lacks classical small-molecule allosteric regulation but is instead controlled through isozyme specialization and sophisticated post-translational mechanisms. In *S. cerevisiae*, two paralogous genes, HMG1 and HMG2, encode isozymes (Hmg1p and Hmg2p) that share ~60% sequence identity with human HMGR and adopt a conserved class I HMGR fold organized as a dimer-of-dimers [29]. While Hmg1p is relatively stable and primarily regulated at the transcriptional level, Hmg2p undergoes regulated degradation via a unique mechanism termed “mallostery,” in which ligand binding induces reversible misfolding [36,37]. This process is mediated by the non-sterol isoprenoid GGPP, which binds to Hmg2p and triggers conformational changes in its transmembrane domain. These changes are recognized by the HMG-CoA reductase degradation pathway (HRD), leading to ubiquitination by the E3 ligase Hrd1p, retrotranslocation from the ER membrane, and subsequent proteasomal degradation [38]. The SSD of Hmg2p is essential for this GGPP-induced misfolding and functions as an intrinsic regulatory module. This process is further modulated by Insig homologs such as Nsg1p, which stabilizes Hmg2p in a sterol-dependent manner by preventing misfolding [37]. Oxysterols derived from sterol biosynthesis act as secondary signals that enhance degradation in cooperation with GGPP [39].

Transcriptional regulation of fungal HMGR is coordinated by sterol-responsive paralogous zinc-cluster transcription factors such as Upc2 and Ecm22, which bind sterol regulatory elements (SRE) in the promoters of *erg* genes and activate expression under sterol depletion [40]. In addition, basal expression of HMG1 depends on the heme-activated regulator Hap1, which functions cooperatively with the Upc2/Ecm22 binding site in the *HMG1* promoter. Upon sterol depletion, Upc2 can activate *erg* gene transcription independently of Hap1 [41].

Alternative regulatory strategies are observed in other fungi. In *Schizosaccharomyces pombe*, HMGR (*hmg1*) is regulated post-translationally through phosphorylation rather than degradation. The Insig homolog Ins1 promotes phosphorylation of conserved residues (Ser1024 and Thr1028) in the catalytic domain in a process dependent on the stress-activated MAP kinase Sty1/Spc1. This modification increases the *K*_m_ for NADPH and reduces catalytic activity without affecting protein levels, linking HMGR function to nutrient status and stress conditions such as osmotic stress or glucose limitation [42,43].

In contrast to eukaryotic Class I HMGRs, certain bacteria such as *Pseudomonas mevalonii*, isolated from soil, possess Class II HMGR enzymes adapted for metabolic flexibility. This organism can utilize mevalonate as a sole carbon source by reversing the canonical pathway and oxidizing mevalonate to HMG-CoA using NAD^+^ instead of NADPH [44,45]. Structurally, Class II HMGRs lack the N-terminal transmembrane domain and consist primarily of the catalytic region, sharing only 14–20% sequence identity with Class I enzymes despite conserving key active-site residues [46]. Together, these diverse regulatory mechanisms highlight HMGR as a central metabolic control point, integrating transcriptional, post-transcriptional, and post-translational signals to finely tune isoprenoid biosynthesis across different domains of life.

### 2.2. The Lower Mevalonate Pathway: From Mevalonate to Isoprenoid Precursors, Variants

Steps 4–6: Mevalonate Phosphorylations and Decarboxylation.

The lower mevalonate pathway converts (R)-mevalonate into the activated isoprene units IPP and DMAPP through three successive ATP-dependent enzymatic phosphorylation reactions. However, following mevalonate formation, the pathway diverges across domains of life. In eukaryotes, mevalonate undergoes two sequential phosphorylation at the 5-OH group. The first reaction is catalyzed by mevalonate kinase (MVK; *erg12*; EC 2.7.1.36), producing mevalonate-5-phosphate (MVAP). This is followed by a second phosphorylation mediated by phosphomevalonate kinase (PMK; *erg8*; EC 2.7.4.2), yielding mevalonate-5-diphosphate (MVAPP) [28,32,47,48,49,50,51,52,53].

**Figure 5:**
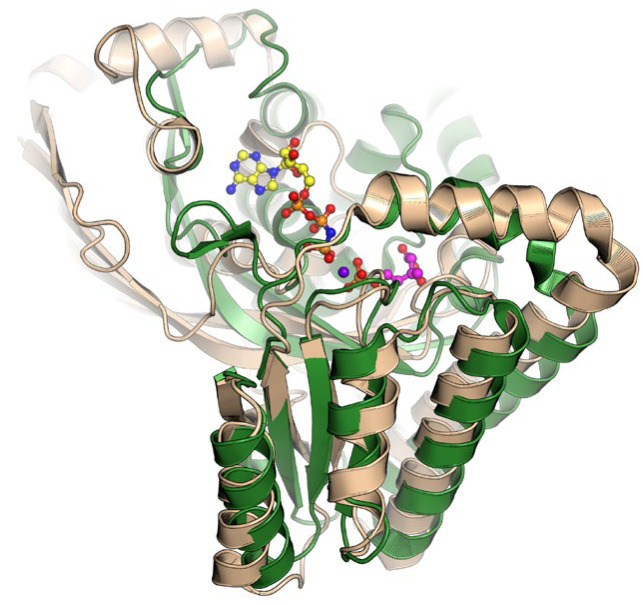
Structure and mechanism of mevalonate kinase (MVK) and phosphomevalonate kinase (PMK). Structural superposition of mevalonate kinase from *Homo sapiens* (beige) and phosphomevalonate kinase from *Streptococcus pneumoniae* (green), represented in cartoon. The region with the highest structural similarity is shown in the foreground. Phosphomevalonate (magenta) and the non-hydrolysable ATP analog AMPPNP (yellow) are shown as observed in the phosphomevalonate kinase structure. PDB entries 2R3V and 3GON.

About 13 MVK crystal structures have been deposited in the PDB (search up to 04/14/2026) from bacteria (one), eukaryote (six) and archaea (six). Only two high-resolution crystal structures (≤2.0 Å) are derived from the eukaryote *Leishmania major* (one) and the archaeon *Methanosarcina mazei Go1* (one). The crystal structure of human MVK (Figure 5) reveals a homodimeric assembly [54], whereas no structure of a fungal ortholog has been reported to date. MVK does not have the rate-limiting properties of HMGR, but it has been shown that its activity is regulated via feedback inhibition by intermediates in the isoprenoid/cholesterol pathway, including GPP, FPP and GGPP [47]. In fungi, MVK is predominantly localized in the cytosol, and its activity is regulated at multiple levels. At the transcriptional level, expression of the *erg12* gene is tightly controlled through sterol-responsive regulatory elements, enabling feedback regulation by pathway end products. In addition, *erg12* mRNA levels can be modulated by environmental conditions and are often upregulated in response to antifungal agents [41]. At the post-translational level, MVK activity is modulated by substrate availability, particularly mevalonate, as well as by feedback inhibition from downstream isoprenoid intermediates. In particular, FPP and GGPP act as potent competitive inhibitors compared to ATP [54,55,56].

Members of the GHMP kinase superfamily, MVK and PMK, catalyze ATP-dependent phosphoryl transfer reactions through a conserved direct in-line mechanism. In both cases, the substrate hydroxyl or phosphate group performs a nucleophilic attack on the γ-phosphate of ATP, proceeding via a pentacoordinate transition state and yielding ADP as a coproduct. For MVK, the C_5_ hydroxyl group of mevalonate acts as the nucleophile, generating mevalonate-5-phosphate (MVAP). In PMK, a non-bridging oxygen of the 5-phosphate group of MVAP serves as the nucleophile in a second phosphorylation step, producing mevalonate-5-diphosphate (MVAPP). These reactions are strictly dependent on divalent metal ions, typically Mg^2+^, which coordinate ATP and stabilize the accumulation of negative charge in the transition state. Structural and kinetic studies indicate that substrate binding induces conformational rearrangements characteristic of the GHMP fold, promoting precise alignment of ATP and the acceptor group within the active site. This induced fit ensures efficient catalysis and stereospecific phosphoryl transfer across both enzymatic steps [4,51,54,57,58,59].

Structural studies of PMK have deposited five crystal structures in the PDB (search up to 14 April 2026) from bacteria (three) and eukaryote (two). Three high-resolution crystal structures (≤2.0 Å) are derived from the bacterium *Streptococcus pneumoniae R6*, the insect *Bombyx mori*, and *Homo sapiens*. Structural studies indicate that PMK undergoes substrate-induced conformational changes that promote proper alignment of ATP and MVAP within the active site, facilitating efficient phosphoryl transfer. Conserved residues within the GHMP kinase fold participate in ATP binding and positioning, as well as in stabilizing the transition state. Unlike some kinases that form covalent intermediates, PMK follows a sequential ordered mechanism without formation of a phosphorylated enzyme intermediate. This catalytic step is essential for channeling carbon flux toward the production of IPP, the universal precursor of isoprenoids. Interestingly, PMK occurs in two distinct, non-orthologous forms. One form, orthologous to the *S. cerevisiae erg8* gene, is present in eubacteria, fungi, and plants. In contrast, orthologous of the human PMK gene are largely restricted to animals [4,59,60]. Human PMK activity is primarily regulated at the transcriptional level by SREBP-1 and SREBP-2, leading to increased expression under sterol-depleted conditions. Additionally, the gene is subject to hypoxic repression [40,61,62].

**Figure 6:**
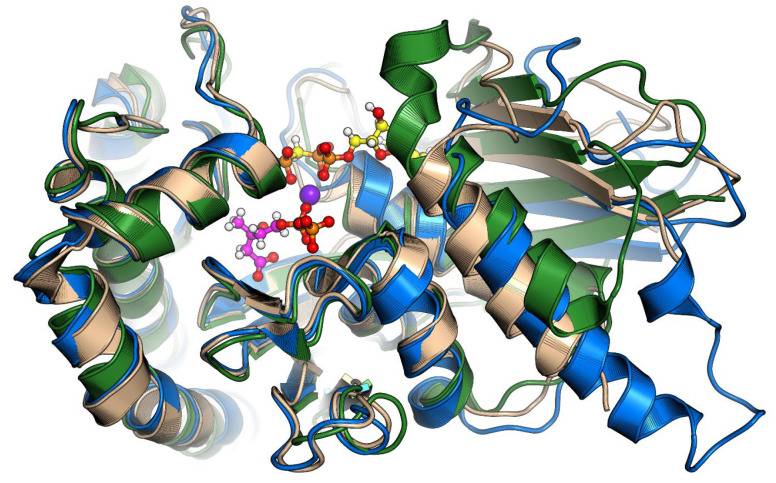
Structure and mechanism of mevalonate diphosphate decarboxylase (MDD). Structural superposition of *Homo sapiens* (beige), *Saccharomyces cerevisiae* (blue), and *Enterococcus faecalis* (green) mevalonate diphosphate decarboxylase, represented in cartoon. The human and yeast structures are in the apo form, while mevalonate 5-diphosphate (MVAPP; magenta) and the non-hydrolysable ATP analog AMPPCP (yellow) are shown as observed in the *E. faecalis* structure. PDB entries 3D4J, 1FI4 and 6E2U.

The MVAPP undergoes an ATP-dependent decarboxylation by mevalonate diphosphate decarboxylase (MDD; *erg19*; EC 4.1.1.33) to yield IPP (Figure 1) [28,32,47,48,49,50,51,52,53]. In the PDB (search up to 14 April 2026) 36 crystal structures have been deposited from bacteria (26), eukaryotes (six), and archaea (four). Fifteen high-resolution crystal structures (≤2.0 Å) are derived from bacteria (10), eukaryotes (three), and archaea (two). MDD is a cytosolic homodimeric enzyme (Figure 6) that catalyzes the ATP-dependent, Mg^2+^-requiring decarboxylation of MVAPP to produce isopentenyl diphosphate [63,64]. In fungi, transcriptional regulation of MDD is primarily by the transcription factors *Upc2p* and *Ecm22p*, which bind sterol regulatory elements (SREs) and upregulate expression under conditions of sterol depletion [40,61].

MDD is also classified within the GHMP kinase ATP-dependent superfamily, based on its conserved structural fold and its use of ATP to phosphorylate the substrate. However, it is functionally distinct, as it catalyzes the ATP-dependent decarboxylation of MVAPP to produce IPP, CO_2_, and ADP. This reaction is unique within the pathway, as it couples phosphorylation, decarboxylation, and dehydration-like elimination into a single catalytic cycle, thereby generating the first activated isoprene unit (IPP), which serves as the universal precursor for all downstream isoprenoid biosynthesis [63,64,65]. The reaction proceeds through an initial phosphorylation, in which the γ-phosphate of ATP is transferred to the C_3_-hydroxyl of MVAPP, generating a transient and unstable 3-phospho-mevalonate-5-diphosphate intermediate. This activated intermediate undergoes decarboxylation followed by elimination (β-elimination-like decarboxylation) that forms the isoprenoid double bond characteristic of IPP. Thus, ATP is not used for net phosphorylation of the final product but instead serves to activate the substrate for decarboxylation. Divalent metal ions, typically Mg^2+^, are required for catalysis, coordinating ATP and stabilizing negative charges that develop during the transition states.

Structural and biochemical studies indicate that substrate binding induces conformational changes that properly orient ATP and MVAPP within the active site, facilitating both phosphorylation and subsequent decarboxylation [64,65,66]. Thus, although it shares evolutionary and structural features with GHMP kinases, MDD performs a more complex reaction that extends beyond simple phosphoryl transfer and is often considered a specialized or divergent member of the family [4,66].

Archaea exhibit at least two modified variants of the MVA pathway that differ in the sequence of phosphorylation and decarboxylation steps (Figure 1) [4,66,67,68]. In *Haloferax volcanii*, the archaeal MVA pathway I (Figure 1) involves a single phosphorylation of mevalonate at the C_5_ hydroxyl group, followed by decarboxylation to form isopentenyl phosphate (IP), and a final phosphorylation step that yields IPP [66]. In contrast, the archaeal MVA pathway II variant (Figure 1), identified in *Thermoplasma acidophilum*, begins with phosphorylation at the C_3_ hydroxyl group, followed by a second phosphorylation at the C_5_ position to produce mevalonate-3,5-bisphosphate (MVABP). This intermediate is subsequently decarboxylated to IP and then phosphorylated to generate IPP [67,68].

Steps 7: Isomerization IPP-DMAPP.

Before participating in other subsequent condensation reactions, IPP must be partially isomerized to its more reactive allylic isomer, DMAPP (Figure 1). This reversible interconversion is catalyzed by type I isopentenyl diphosphate isomerase (IDI1; EC 5.3.3.2), an enzyme in eukaryotes and bacteria that is essential for isoprenoid biosynthesis [69,70,71,72,73]. In *S. cerevisiae*, IDI1 is a cytosolic 33 kDa monomeric enzyme that catalyzes the reversible conversion between IPP and DMAPP [69,72]. Consistent with its essential role, IDI1 is encoded by a single-copy gene, and its disruption is lethal, as demonstrated by tetrad analysis of heterozygous diploid mutants [74]. Contrary to several other mevalonate pathway enzymes subject to sterol-mediated transcriptional feedback, IDI1 exhibits largely constitutive expression with minimal transcriptional modulation across physiological conditions, and no allosteric regulators or pathway-intermediate feedback inhibitors have been identified [72].

**Figure 7:**
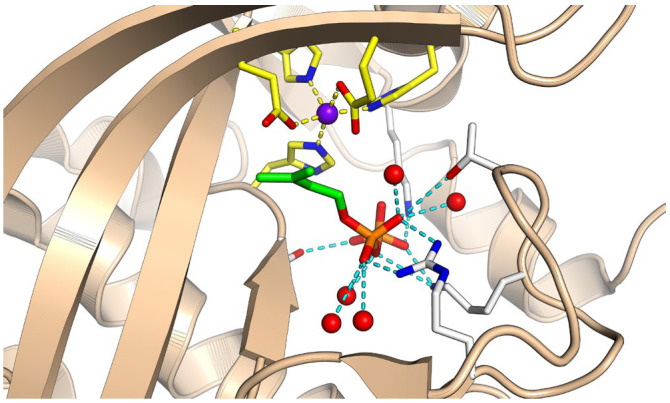
Structure and mechanism of type-1 isopentenyl diphosphate isomerase (IDI1). Crystal structure of active site from isopentenyl diphosphate isomerase from *Homo sapiens* in complex with the substrate analog ethanol amine pyrophosphate (EAPP). The phosphate groups of EAPP are coordinated by residues shown as white sticks and by water molecules (red spheres). The catalytic manganese ion (Mn^2+^, purple sphere) is coordinated by residues depicted in yellow. PDB entry 2ICK.

Although the crystal structure of human IDI1 has been solved (Figure 7), no structure of a fungal ortholog has yet been deposited in the Protein Data Bank. The structure of human IDI1 has provided detailed insights into substrate binding, metal coordination, and the positioning of catalytic residues, supporting a stepwise mechanism involving protonation, carbocation rearrangement and deprotonation. This mechanism distinguishes IDI1 from type II IDI enzymes, which instead employ a flavin-dependent radical-based mechanism. IDI1 belongs to the type I IDI family, which requires a divalent metal cofactor, typically Mn^2+^ or Mg^2+^, to achieve its catalytically active conformation (Figure 7) [70].

The reaction occurs through a protonation–deprotonation mechanism that involves the transient formation of a carbocation intermediate. Specifically, the enzyme first protonates the C4-C5 double bond of IPP to generate a tertiary carbocation, followed by deprotonation at C_2_ to produce DMAPP with the shifted double bond. The metal ion coordinates the diphosphate moiety of the substrate, stabilizing negative charge and properly orienting IPP within the active site. Conserved acidic and basic residues act as general acid/base catalysts, mediating proton transfer during the isomerization. Structural and mechanistic studies indicate that the enzyme stabilizes the high-energy carbocation intermediate through electrostatic interactions and precise active-site geometry, ensuring both efficiency and stereochemical control. The reaction is fully reversible and occurs without the formation of covalent enzyme-substrate intermediates [4,70,72].

### 2.3. The Methylerythritol Phosphate (MEP) Pathway: An Alternative Route to Isoprenoid Biosynthesis

Most eubacteria, plant plastids, and several photosynthetic eukaryotes (including unicellular green algae and other photosynthetic protists, as well as the malarial parasite *Plasmodium falciparum*), synthesize isoprenoids through an alternative pathway known as the 2-C-methyl-D-erythritol 4-phosphate (MEP) pathway, also referred to as the non-mevalonate pathway. This pathway was first identified in eubacteria in the 1990s [75] and produces IPP and DMAPP from D-glyceraldehyde-3-phosphate (G3P) and pyruvate [1,8,76,77,78]. In contrast to the MVA pathway, which derives these intermediates from acetyl-CoA, the enzymatic steps of the MEP pathway are completely distinct.

The evolutionary origins and taxonomic distribution of isoprenoid biosynthesis pathways remain complex and are still under active discussion. The MEP pathway represents the predominant pathway for isoprenoid precursor biosynthesis in most bacteria, including *Escherichia coli* and the pathogen *Mycobacterium tuberculosis*, as well as in plastids of plants and algae [8,79,80,81]. In contrast, the MVA pathway is primarily associated with archaea and eukaryotes, although it has also been identified in several bacterial taxa, particularly among Gram-positive Firmicutes and selected Proteobacteria. Furthermore, some bacterial species harbor both the MEP and MVA pathways, suggesting a complex evolutionary history and metabolic diversification of isoprenoid biosynthesis [79,82,83,84,85]. The presence of enzymes in this pathway in certain lineages has traditionally been attributed to horizontal gene transfer (HGT) from archaeal or eukaryotic donors [86,87].

In plants, other photosynthetic eukaryotes, and apicomplexans (a group of parasitic protists derived from a photosynthetic ancestor), both the MVA and MEP pathways coexist. In these organisms, it is assumed that the plastids (either fully functional or highly reduced, as in apicomplexans) acquired the MEP pathway through gene transfer from the ancestral cyanobacterial endosymbiont that gave rise to these organelles [1,88]. However, phylogenetic analyses reveal that some enzymes in this pathway are of cyanobacterial origin, while others appear to derive from non-cyanobacterial lineages [8,89,90,91]. Interestingly, most archaea lack the final three enzymes of the classical (eukaryotic-like) MVA pathway (PMK, MDD, and IDI), as revealed by genomic analyses [7,8,92]. These enzymes are present only in specific archaeal clades and may have been acquired through HGT from bacterial or eukaryotic sources [7].

Overall, each domain of life is characterized by a predominant isoprenoid biosynthesis pathway: the classical MVA pathway in eukaryotes, two variants of the pathways in archaea (I and II) (Figure 1), and the MEP pathway in bacteria. However, evidence suggests that the MVA pathway may be ancestral not only in archaea and eukaryotes, but also in bacteria, challenging earlier assumptions that its presence in bacteria resulted solely from HGT [8]. This raises the possibility that the MVA pathway was already present in the last universal common ancestor (LUCA), implying that primitive cell membranes may have incorporated isoprenoid-derived components. Despite their distinct biochemical steps, both the MVA and MEP pathways converge on the same essential products, IPP and DMAPP. In higher plants, these pathways are compartmentalized: the MEP pathway occurs in plastids, whereas the MVA pathway occurs in the cytosol [75,78].

Detailed structural and mechanistic characterization of enzymes involved in the MVA pathway provides a valuable framework for rational protein engineering, metabolic optimization, and synthetic biology applications, although the successful translation of these insights into improved phenotypes often remains context-dependent and requires empirical validation. Knowledge of catalytic residues, substrate-binding pockets, cofactor interaction sites, oligomerization interfaces, conformational dynamics, and regulatory domains has enabled the design of engineered enzyme variants with enhanced catalytic efficiency, improved thermostability, altered substrate specificity, and reduced sensitivity to feedback inhibition. Such structure-guided strategies are particularly relevant for metabolic engineering approaches aimed at redirecting carbon flux, optimizing precursor availability, and increasing the microbial production of terpenoids, sterols, carotenoids, and other industrially relevant isoprenoids.

Several enzymes in the MVA pathway are associated with the biotechnological potential of structure-based engineering approaches. In the MK enzyme, structural and biochemical studies have identified residues associated with competitive inhibition by downstream isoprenoid intermediates such as FPP and GGPP. Mutations targeting these regulatory regions have been proposed as promising strategies to improve feedback-associated metabolic bottlenecks in engineered microbial hosts [54]. Likewise, the N-terminal membrane-associated regulatory domain of eukaryotic HMGR has been extensively investigated in metabolic engineering through the expression of truncated cytosolic variants that prevent sterol-dependent degradation, thereby substantially increasing MVA-pathway flux and isoprenoid biosynthesis in engineered yeast strains [93]. Structural dynamics have also provided important insights for catalytic optimization. In mevalonate diphosphate decarboxylase (MDD), substrate-induced conformational rearrangements involving the phosphate-binding loop and the β10-α4 loop, which close over the active site during catalysis, represent attractive targets for engineering strategies focused on modulating substrate interactions and catalytic performance [65]. Together, these studies highlight how structural and mechanistic insights into MVA-pathway enzymes can support the rational development of optimized microbial platforms with improved pathway stability, flux control, and isoprenoid productivity.

## 3. Metabolic Outputs of the MVA and MEP Pathways: Diversity of Isoprenoid End Products

IPP and DMAPP are central branch-point metabolites in isoprenoid biosynthesis, participating in a wide variety of condensation reactions that generate structurally and functionally diverse isoprenoid compounds. These metabolites are also involved in multiple essential biochemical processes, including the prenylation of specific tRNA species, as well as the biosynthesis of sterols, carotenoids, and longer-chain polyisoprene diphosphates required to produce dolichols and quinones (including ubiquinone) (Figure 1) [4,28,94,95]. Thus, IPP contributes to the isopentenylation of specific tRNA species, such as those carrying Ser, Phe, Leu, Cys, and Trp, through the transfer of a five-carbon isoprene unit derived from IPP (Figure 1) [94,95]. Additionally, in eukaryotes, DMAPP can be dephosphorylated and recycled into acetyl-CoA or acetoacetate in mitochondria, providing metabolic flexibility and preventing accumulation of excess isoprenoid intermediates (Figure 1) [96].

The biosynthesis of terpenoids in plants and microorganisms occurs mainly through the MVA and MEP pathways, which produce the universal five-carbon precursors, IPP and DMAPP [97,98]. These isomeric precursors (C5) are subsequently condensed by prenyltransferases (PTs) to generate allylic diphosphate intermediates of varying chain lengths, such as geranyl (C10), farnesyl (C15), geranylgeranyl (C20), geranylfarnesyl diphosphate (C25) and squalene (C30) (Figure 1) [99,100]. These intermediates act as substrates for terpene synthases (TSs), which convert these precursors into isoprenoids by condensation, isomerization and conjugation reactions. TSs are a diverse class of enzymes traditionally classified as type I and II, according to their mechanism of action. Type I TSs catalyze the metal-dependent ionization of a diphosphate to generate a reactive allylic carbocation on the terpene skeleton. In contrast, type II TSs initiate cyclization via the protonation of an alkene or epoxide moiety, yielding a stable tertiary carbocation intermediate [101,102,103]. TSs have the ability to form multiple products from a single substrate, with varying degrees of specificity, and several are multi-substrate enzymes [104]. Terpenoids can be further modified and functionalized via hydroxylation, oxidation, glycosylation, acylation, acetylation, methylation and esterification by terpenoid-modifying enzymes, such as cytochrome P450s (CYPs), glycosyltransferases and dehydrogenases [105,106,107,108,109,110].

The enzymes responsible for the conversion of mevalonate to FPP are mainly present in the cytoplasm and have been studied in detail, isolated and thoroughly characterized [69]. At the beginning of the biosynthesis of terpenoids, the farnesyl pyrophosphate (FPP) synthase (*erg20*, EC 2.5.1.10), an enzyme that belongs to the class of prenyltransferases, catalyzes sequential two head-to-tail condensations. The first step condenses a DMAPP molecule with two molecules of IPP to form the intermediate GPP, which then condenses with another IPP molecule to give FPP (Figure 1). The two terminal double bonds in FPP are in the trans configuration [28,111,112,113].

FPP is the last common intermediate in the synthesis of the lipid end products, i.e., cholesterol and dolichol, and subsequent processes are associated with various organelles. FPP is also considered to be the precursor for the biosynthesis of the polyprenyl-PP side chain of ubiquinone (Figure 1). However, as will be discussed below, the data available indicate that DMAPP and GPP may be the preferred allylic substrates for trans-prenyltransferases. FPP is further elongated to all-trans-geranylgeranyl pyrophosphate (GGPP) (Figure 1) in the cytosol and these two isoprenoids (FPP and GGPP) are utilized in cytosolic protein modification reactions [28]. Specifically, two enzymes, farnesyltransferase and geranylgeranyltransferase, are responsible for carrying out the prenylation process in the cell. The process involves the covalent attachment of hydrophobic molecules (either the C-15 isoprene farnesyl or the C-20 isoprene geranylgeranyl groups) to the C-terminal end of some proteins including the γ subunit of heterotrimeric G proteins, heme-A, nuclear lamins and the small GTP-binding proteins [114,115]. Prenylation promotes the attachment of these proteins to internal cell membranes by means of a lipid anchor. Posttranslational modification and activation of GTP-binding proteins Rho, Rac, Rab, Rap, and Ras play important roles in many important signaling cascades within the cell [47,115,116]. The first committed step of hepatic cholesterol biosynthesis is catalyzed by squalene synthase (SS), which converts FPP into squalene (Figure 1). Squalene is then transformed into lanosterol through a two-step process involving epoxidation and cyclization. Subsequently, lanosterol undergoes a series of approximately 19 enzymatic reactions to yield cholesterol [52].

## 4. Functional Significance of the Mevalonate Pathway Across Kingdoms, with Emphasis on Fungal Biology

### 4.1. Biological Roles of the Mevalonate Pathway in Fungi

In fungi, the MVA pathway plays a central role in cellular physiology by supplying precursors for the biosynthesis of sterols, isoprenoids, and other essential metabolites. One of its most important functions is the production of ergosterol, the principal sterol component of fungal membranes. In this pathway, IPP-derived intermediates are converted into ergosterol via FPP, squalene, and lanosterol. Ergosterol biosynthesis is a major target for antifungal drugs, with resistance often arising from mutations in pathway genes [117]. It is a functional analog of cholesterol in fungi and differs structurally by the presence of two additional double bonds and a methyl group. Like cholesterol in animal cells, ergosterol is essential for membrane integrity, fluidity, and proper cellular function, making its biosynthetic pathway a major target of antifungal drugs, including azoles and polyenes [52,118,119,120]. Consequently, the fungal MVA pathway represents a critical metabolic hub that links primary metabolism to membrane homeostasis, stress responses, and environmental adaptation.

Beyond sterol biosynthesis, fungi also produce bioactive terpenes, yielding anti-microbials and molecules with valuable pharmacological properties [121]. Pathogenic species also rely on the mevalonate pathway for biosynthesis of iron-chelating siderophores, which play important roles in virulence and ecological interactions [122]. In addition, yeasts are widely used as platforms for metabolic engineering to produce valuable isoprenoids, including limonene (a flavor and fragrance compound), farnesene (a biofuel precursor), and artemisinic acid (antimalarial drug) [123].

Protein prenylation is also required for the proper localization and activity of numerous regulatory proteins involved in signaling pathways, cytoskeletal organization, vesicular trafficking, and polarized growth. Consequently, protein prenylation plays a critical role in fungal morphogenesis, hyphal extension, pathogenicity, and developmental processes [124,125,126].

Accumulating evidence indicates that the MVA pathway also contributes to fungal adaptation to environmental stresses and developmental regulation. Alterations in sterol composition and isoprenoid metabolism have been associated with responses to oxidative and osmotic stress, temperature fluctuations, and exposure to antifungal agents [117,120,127]. Furthermore, the pathway influences sporulation, filamentation, and other developmental transitions that are essential for fungal survival and ecological fitness. Collectively, these findings highlight the MVA pathway as a multifunctional metabolic network that coordinates membrane biogenesis, cellular signaling, stress responses, and developmental processes in fungi.

### 4.2. Cross-Kingdom Distribution and Functional Diversity of the Mevalonate Pathway

The MVA pathway exhibits remarkable diversity across biological kingdoms. Animals and fungi depend exclusively on this pathway for isoprenoid biosynthesis; whereas, most bacteria, plant chloroplasts, and green algae utilize the alternative MEP pathway [78].

In animal cells, sterol-derived isoprenoids, such as cholesterol, play essential roles in cell cycle regulation, membrane structure and fluidity, and cellular signaling [43,128]. The conversion of HMG-CoA to MVA by HMG-CoA reductase represents the key regulatory step of the pathway and is tightly controlled by cholesterol-mediated feedback inhibition that prevents the excessive accumulation of sterols and their intermediates. This enzyme is the primary target of statins, a class of competitive inhibitors widely used in clinical practice to lower cholesterol levels and in treatment of cardiovascular disease [43,129].

Beyond sterols, non-sterol isoprenoids play critical roles in post-translational protein modification, regulation of gene expression, and cytoskeletal organization [52]. They also contribute to the biosynthesis of essential metabolites, such as ubiquinone and carotenoids, which are involved in electron transport and antioxidant defense [130]. These functions underscore the central role of the MVA pathway in cellular homeostasis and regulation, including processes associated with cancer, inflammation, oxidative stress, and hormone biosynthesis [131].

In plants, according to studies in *Arabidopsis thaliana*, both pathways coexist but are compartmentalized: MVA enzymes are localized in the cytosol, endoplasmic reticulum, and peroxisomes; whereas, MEP pathway enzymes act in plastids. These pathways are not functionally interchangeable, as inhibition of one cannot be fully compensated by the other, despite evidence of metabolite exchange between compartments [118]. Together, they contribute to the biosynthesis of plant hormones such as brassinosteroids, cytokinins, gibberellins, and abscisic acid, which regulate seed and plant development, stress responses, fertility, reproduction, differentiation, senescence, and fruit development. Nevertheless, these two pathways form a complex and intertwined metabolic system that is continuously controlled at various levels [1,118,119].

Plant hormones derived from isoprenoid intermediates can be synthesized within the plant metabolism or by pathogens and the soil microbiome. Since the MVA and MEP pathways are unequally distributed across kingdoms, the crosstalk between these organisms and pathways is a promising subject of study. The species present in the microbiota influence plant isoprenoid status, and the contrary is also true: plants with deficient isoprenoid production are colonized by different bacterial species [124]. Considering that terpenoids are used in the chemical communication between plants and other organisms, it makes sense that they are used to modulate the rhizosphere to favor microorganisms involved in plant defense [125].

Isoprenoids in plants also function as photosynthetic pigments, defense compounds, and signaling molecules. Many, including carotenoids, menthol, and paclitaxel, have significant industrial applications in pharmaceuticals, cosmetics, agriculture, and food industries [131,132]. Additionally, plant-microbiome interactions are influenced by isoprenoid metabolism, with terpenoids playing key roles in rhizosphere communication and microbial recruitment [126,127].

Although most bacteria rely on the MEP pathway, some Gram-positive cocci species possess a functional MVA pathway, where intermediates contribute to cell wall biosynthesis and virulence [87], as demonstrated in *Staphylococcus aureus* [133]. In archaea, modified versions of the MVA pathway generate isoprenoid chains that, like glycerol phosphate, are essential for unique membrane architectures that support survival under extreme conditions at high temperatures [1,67].

## 5. Biotechnological Applications of Isoprenoid Biosynthesis Pathways

### 5.1. Intermediate Metabolites Mevalonate and Mevalonate Phosphate as Molecules of Industrial Interest

In synthetic biology, the reassignment of intermediate metabolites as end products was a paradigm shift in industrial biotechnology. Molecules such as MVA and MVAP were previously considered only transient intermediates in the isoprenoid synthesis pathway. However, these pathways have been modified through metabolic engineering which has promoted changes in these pathways, transforming these intermediates into high-value commercial molecules [88,134,135,136].

MVA is no longer considered exclusively as a biological precursor, but rather a central intermediate in the isoprenoid pathway. Furthermore, it is now known as a high-value “platform molecule” due to its chemical stability and the ease with which recombinant microorganisms can be modified to accumulate it in high concentrations [135,136,137,138]. One of the main applications of MVA is in the cosmetics industry, where topical mevalonic acid is used to stimulate *de novo* cutaneous cholesterol synthesis and maintain epidermal barrier homeostasis in aging skin [137]. The industrial viability of this molecule is mainly due to advances in synthetic biology, which resulted in record production in *E. coli*, reaching 111.3 g/L in fed-batch processes [135,136]. Furthermore, the innovative use of autotrophic hosts, such as *Cupriavidus necator*, has demonstrated the possibility of converting CO_2_ and H_2_ into mevalonate, reaching titters of approximately 10 g/L, which represents a milestone in the production of C6 compounds from renewable C1 carbon sources [137].

Besides MVA, MVAP and other phosphorylated intermediates are emerging as products of strategic interest, mainly in the development of pathways to overcome barriers such as the metabolic toxicity of IPP [139]. Metabolic engineering strategies targeting “bypass pathways” consider MVAP as the main substrate to produce five-carbon alcohols, such as isopentenol, which exhibits superior combustion properties compared to ethanol for biofuels applications [139,140,141]. This approach not only mitigates inhibitory bottlenecks in cellular growth but also optimizes the energy efficiency of the biotechnological process, reducing ATP demand and increasing large-scale productivity [139,142].

The transition from laboratory production to the industrial scale of intermediate compounds and the determination of scale-up parameters are essential for the economic viability and success of commercial production. A recent study on the use of parallel bioreactors to perform multiple simultaneous fermentations was proposed. These parameters are fundamental for controlling cell growth rate, biomass formation, cell maintenance, and the production of the compound of interest. This approach allows for biotechnological production process design and optimization and facilitates rapid and economical commercial feasibility studies [136,143].

### 5.2. Production of Biopharmaceuticals and Biomaterials

The MVA and MEP pathways constitute the biosynthetic basis to produce the largest and most diverse class of natural products: terpenoids, which total more than 80,000 identified structures. These compounds, mainly those of high clinical value, such as artemisinin (antimalarial), paclitaxel (anticancer), cannabinoids, and various carotenoids with antioxidant and anti-inflammatory properties [144,145,146] are produced from the precursors IPP and DMAPP. The use of metabolic engineering applied to these pathways can be an alternative to overcome the limitations of natural plant extraction and chemical synthesis, as well as to enable the biotechnological transformation of microorganisms such as *S. cerevisiae* and *E. coli*, and microalgae, into efficient cellular platforms. The use of yeasts, particularly *S. cerevisiae*, has enabled significant advances in the production of terpenoid through the MVA pathway. Detailed knowledge of this well-regulated endogenous MVA pathway, as well as the classification of this microorganism as a GRAS (Generally Recognized As Safe), has transformed it into an important cellular platform to produce molecules of industrial and biotechnological interest [147,148,149]. Unconventional yeasts, such as *Yarrowia lipolytica* and *Komagataella phafii*, are also being used as cell platforms due to specific characteristics such as the ability to generate acetyl-CoA and NADPH, molecules important in isoprenoid biosynthesis, as well as their high efficiency in the secretion and glycosylation of heterologous proteins [150,151,152,153,154].

In these microorganisms, and also in bacteria, metabolic engineering consists of integrated strategies of overexpression of key genes in the MVA pathway, increasing the availability of precursors (such as HMG-CoA reductase) and metabolic modulation to maximize the yield of the desired product. Subcellular compartmentalization engineering is an innovative approach that promotes the relocation of enzymes or entire pathways to specific organelles, such as mitochondria or peroxisomes, thereby allowing the creation of metabolic microenvironments with high substrate concentrations and less interference from competing pathways, resulting in a significant increase in productivity [135,138,140,145,155,156]. Table 1 shows some metabolic engineering strategies in the MVA pathway in yeast.

Metabolic engineering has also used microalgae as an alternative chassi organism to produce biopharmaceuticals and biomaterials [157,158]. Cellular platforms such as *Phaeodactylum tricornutum* and *Chlamydomonas reinhardtii* are being optimized through the DBTL (Design–Build–Test–Learn) cycle and genome-scale metabolic modeling for the sustainable synthesis of complex carotenoids, such as fucoxanthin and astaxanthin [158,159]. Furthermore, since abiotic stress (such as UV-B radiation, drought, and extreme temperatures) are potent inducers of secondary metabolite biosynthesis, the management of cultivation conditions as a “natural” bioengineering tool has also been considered [157]. It is important to highlight that the integration of these synthetic biology technologies with in situ product recovery processes is applicable to the large-scale and carbon-neutral production of bioactive compounds essential for human health [144,158,160].

**Table 1:** Metabolic engineering strategies to enhance mevalonate pathway flux and isoprenoid production.

Strategy/Target	Microorganism	Example Outcome	References
HMGR (tHMGR) over-expression	*Saccharomyces cerevisiae*	431-fold increase squalene; triter-pene boosts	[93,161]
Combinatorial MVA gene overexpression. (*erg10*, *erg13*, *erg12*, etc.)	*Saccharomyces cerevisiae*	>13-fold increase amorpha 4,11 diene	[162,163]
CRISPR multiplex knockouts	*Saccharomyces cerevisiae*	41-fold increase mevalonate without overexpression	[93,164]
Carbon and acetyl CoA rewiring	*Saccharomyces cerevisiae* and *Yarrowia lipolytica*	Up to 101 g/L mevalonate	[161,165,166]

Although metabolic engineering strategies and cellular platforms are being developed and integrated, the toxicity of intermediates and products, the metabolic overload associated with gene overexpression, and the limitation of cofactors (such as NADPH) in the MVA pathway, as well as the structural complexity of various terpenoids, constitute critical barriers to maximizing yields. In this context, advances in next-generation cells that encompass multiple and efficient metabolic regulation strategies, the search for alternative biosynthetic metabolic pathways, and the application of biocatalysis and green chemistry processes could expand the potential for the producing of innovative compounds, making the MVA pathway a benchmark in the sustainable production of high-value-added biopharmaceuticals and biomaterials [135,137,138,156,167].

### 5.3. Drug Production: Statins, Artemisinin, and Other Bioactive Compounds Derived from Terpenoids

The production of drugs derived from terpenoids is one of the most dynamic fields of pharmaceutical biotechnology, driven by the extensive structural diversity and bioactivity of these molecules, which act as anticancer, anti-inflammatory, antimicrobial, and hypolipidemic agents [146]. Terpenoids constitute the largest and most diverse class of plant secondary metabolites, which are synthesized from isoprene (C5) units through the MVA and MEP pathways [146,168]. Among the most clinically relevant compounds derived from this class, statins stand out, such as lovastatin and compactin (first-generation statins), originally isolated from fungi (*Aspergillus terreus* and *Penicillium citrinum*), which act as competitive inhibitors of the HMG-CoA reductase enzyme in the control of hypercholesterolemia [169,170,171,172]. Recent advances in metabolic engineering have allowed the optimization of these strains, using the CRISPR system and adaptive evolution to increase yield and reduce undesirable byproducts [146,170,171]. The production of second-generation statins, such as pravastatin, has shifted from a two-step bioconversion process to single-step fermentative systems [171,173] through metabolic reprogramming of the fungus *Penicillium chrysogenum.* In these systems, the compactin pathway and an evolved cytochrome P450 (CYP105AS1) were introduced to perform the necessary specific hydroxylation, resulting in pilot-scale production levels exceeding 6 g/L [173]. For third-generation statins (e.g., atorvastatin), the current trend is chemoenzymatic synthesis, which uses biocatalysts such as carbonyl reductase and halohydrin dehalogenase (HHDH) under “green chemistry” conditions, eliminating laborious chemical protection and deprotection steps [171].

Meanwhile, artemisinin, a sesquiterpene lactone extracted from *Artemisia annua*, has become established as the main antimalarial treatment due to its effectiveness against the *Plasmodium* spp. parasite [168,174,175]. This pharmacological application of semi-synthetic artemisinin represented a milestone in synthetic biology by genetically modifying the yeast *S. cerevisiae* to produce the stable precursor artemisinic acid in high concentrations, which is subsequently chemically converted into the final drug [174,176]. The heterologous production of other high-pharmacological-value terpenoids via microbial fermentation still faces challenges. The diterpene paclitaxel (Taxol), essential in cancer therapy, and β-elemene, a sesquiterpene from *Curcuma wenyujin* recognized for its ability to induce apoptosis and inhibit mitosis in various tumor cell lines, are under investigation for production using the MVA pathway in microorganisms to ensure greater purity and lower cost [146,168,170,174,177,178]. Additionally, studies with *Astragalus membranaceus* and *Tripterygium wilfordii* have been developed to elucidate key genes for the heterologous synthesis of saponins and diterpenoids, such as triptolide, aiming at applications in immunomodulation and autoimmune diseases [179,180].

The industrial production of these bioactive compounds presents limitations due to the complexity of their biosynthetic pathways in plants, which frequently results in instability in industrial supply and consequently, in price fluctuations [146,168,170,178,179]. To overcome these limitations, pharmaceutical biotechnology has advanced in the use of microorganisms as platforms for the overproduction of molecules and has adopted synthetic biology strategies that allow the reconstruction of metabolic pathways in well-characterized heterologous hosts, such as *S. cerevisiae* and *K. phafii* [170,174,179,181]. Innovations in metabolic engineering, such as the deletion of the gedC gene to eliminate competitive byproducts and the overexpression of membrane proteins (such as TapA) to optimize drug efflux, have significantly increased industrial yields [146,170,171,181]. Another limitation in the production of pharmaceutical terpenoids is the low solubility and high toxicity of many of these compounds. In this case, it is necessary to develop advanced delivery systems, such as nanotransporters, liposomes, and phospholipid complexes, as strategies to improve bioavailability and tissue targeting [180,182,183]. Therefore, the drug production from terpenoids involves the consolidation of robust biocatalysts, the expansion of heterologous synthesis to new cellular platforms, and the adoption of sustainable manufacturing technologies [171,179]. These advances not only guarantee a stable and sustainable supply of essential medicines but also pave the way for the discovery of new therapeutic candidates from the various modified terpene structures [171].

### 5.4. Production of Carotenoids as Nutraceutical Supplements and Cosmetic Active Ingredients

Carotenoids are natural compounds synthesized by photosynthetic and some non-photosynthetic organisms, such as plants, algae, cyanobacteria, bacteria, fungi, and yeast [184,185,186]. They are widely distributed in nature, exhibiting colors in vivo such as red, orange, and yellow. These molecules play an important role in biological systems, acting as photoprotective agents, antioxidants, color attractants, and precursors of plant hormones [184,185,187]. Animals are unable to synthesize carotenoids and, considering their importance for human health and nutrition, they are generally obtained through diet or supplementation. Some of these compounds act as important precursors of vitamin A, as well as antioxidants, strengthen the immune system, and contribute to reproductive processes [184,185,188,189,190,191,192].

These compounds are isoprenoids derived from universal C5 precursors, IPP and DMAPP. More than 1200 carotenoids have been identified in nature to date [193,194] and are classified as tetraterpenes (C40), formed through the condensation of eight isoprene units [184,195]. This biosynthesis occurs via MVA pathway, in the cytosol, and MEP pathway, in the plastids. The products are divided into two primary groups: the carotenes (hydrocarbons), which are most commonly β-carotene, α-carotene, and lycopene; and the xanthophylls (oxygenated carotenoids), which are most commonly lutein, zeaxanthin, and β-cryptoxanthin compounds [184,187,195].

Due to their importance to human and animal health, carotenoids have wide applications in the food, feed, nutraceutical, pharmaceutical, and cosmetic industries. They are also commonly found in products such as dairy products (e.g., fortified milk, yogurt, and butter), beverages (e.g., juices and functional drinks) [196], animal feed for poultry and aquaculture (e.g., pigmentation of egg yolks, skin, and fish flesh), dietary supplements in capsule or oil, pharmaceutical formulations with antioxidant and provitamin A activities, and cosmetic products such as creams, lotions, and sunscreens [194]. However, most commercially available carotenoids are produced by chemical synthesis, given that extraction from natural sources involves high costs and low yields [185,197]. Therefore, alternative carotenoid production strategies based on microbial hosts and biosynthetic processes are crucial to achieving industrial sustainability and scalability [198].

In this context, microorganisms have been frequently used for carotenoid production, integrating metabolic engineering and synthetic biology. The hosts such as *E. coli*, *S. cerevisiae*, and *Y. lipolytica* have been widely used due to their high potential for large-scale production and reduced cost [198,199,200,201]. Yang and Gou, in 2014, engineered an *E. coli* strain *YJM45*, introducing a hybrid MVA pathway to produce β-carotene from glycerol under aerobic fed-batch fermentation conditions [199]. The production of this compound increased 66-fold compared to the control strain (*E. coli JM39*). In another study, a *Y. lipolytica* strain was engineered through MVA overexpression (HMG, *erg13*, and GGS1 genes) and with carotenoid biosynthetic genes (*carB* and *carP*). The strategy combining genetic engineering with culture optimization resulted in a 24-fold increase in production compared to the parental strain (*Y. lipolytica XK2*) [200].

Lycopene is a carotene used as a natural colorant and antioxidant in the food, pharmaceutical, and cosmetic industries. Lycopene production in *E. coli* through metabolic engineering using the heterologous MVA pathway, optimized lycopene biosynthetic genes (*crtE*, *crtB*, *crtI*), and fed-batch fermentation (100 L) resulted in a 10-fold increase in production compared to the control strain with the endogenous MEP pathway [202]. In conclusion, MVA pathway engineering is crucial for increasing precursors IPP and DMAPP, and metabolic flux, thereby allowing sustainable and scalable production of high-value carotenoids in modern industrial biotechnology.

### 5.5. Isoprenoids as Precursors to Renewable Fuels

The MVA pathway constitutes a versatile biosynthetic platform for the sustainable production of advanced biofuels. Several monoterpenes and sesquiterpenes exhibit physicochemical properties desirable for fuel applications, including high volumetric energy density, low freezing points, enhanced oxidative stability, and performance characteristics comparable to those of petroleum-derived fuels [139,203,204,205].

Although plants are the primary natural source of terpenes [206], heterologous terpene production via metabolic engineering and synthetic biology in microbial hosts has emerged as a more sustainable and economically viable alternative for industrial-scale biofuel production [207]. Terpene biosynthesis has been successfully implemented in microorganisms such as *E. coli* and yeasts, including *S. cerevisiae* and *Y. lipolytica* [207,208,209]. However, efficient production of mono- and sesquiterpenes critically depends on the intracellular availability of the universal isoprenoid precursors IPP and DMAPP. While *E. coli* natively synthesizes IPP via the methylerythritol phosphate (MEP) pathway, yeasts utilize the MVA pathway, which is generally more efficient. Consequently, *E. coli* is frequently engineered to harbor a heterologous MVA pathway to enhance terpene production yields [207].

Among the most widely produced terpenes are monoterpenes such as limonene and pinene, as well as sesquiterpenes such as farnesene and bisabolene, all of which are valuable intermediates or direct candidates for the production of diesel, jet, and aviation fuels [207,208,210]. As a result, terpene biosynthesis has been extensively investigated. For instance, Rolf et al. (2020) reported limonene production in engineered *E. coli* using glycerol as a carbon source in a bioreactor, achieving a final titer of 3.6 g/L [208]. Tashiro et al. (2016) introduced a pinene synthase along with MVA pathway genes and isopentenyl diphosphate isomerase into *E. coli*, resulting in pinene production of 140 mg/L in shake-flask cultures [211]. Similarly, Zhang et al. (2014) engineered *E. coli* with a sabinene synthase, a GPP synthase, and a heterologous MVA pathway, reaching titer of 82.18 mg/L in shake flasks and 2.65 g/L in bioreactor fermentations [212]. At the industrial level, Amyris Inc. demonstrated the large-scale production of β-farnesene in *S. cerevisiae*, achieving titers of 130 g/L in a 2000 L fermenter [210].

Comparable advances have been reported in *Y. lipolytica*, with β-farnesene production reaching 28.9 g/L in bioreactor systems [209]. Furthermore, Peralta-Yahya et al. (2011) [213] engineered both *E. coli* and *S. cerevisiae* to produce bisabolene, a key precursor for advanced biodiesel, achieving titers of approximately 900 mg/L through the integration of a heterologous MVA pathway and specific sesquiterpene synthases. Together, these studies highlight that metabolic engineering of microbial platforms, either through heterologous implementation or optimization of the MVA pathway, combined with targeted terpene biosynthesis, represents a robust, scalable, and economically promising strategy for producing next-generation biofuels.

## 6. Metabolic Engineering and Bioengineering of Microorganisms for Isoprenoid Production

Traditional extraction of terpenoids from natural resources usually results in unsustainable yields, long grow cycles, and is susceptible to seasonal and geographic changes [144,214]. Therefore, since the advent of microbial biotechnology, various microorganisms have been explored to optimize complex metabolic pathways of industrial relevance, such as the MVA pathway. The use of microbial host organisms has gained significant attention due to advantages over plant or animal extraction and chemical synthesis, including environmental sustainability, cost-effectiveness, and process scalability [214,215,216,217,218]. The combination of synthetic biology tools, a deep understanding of biological systems and metabolic engineering is crucial for future advancements in MVA pathway engineering in microorganisms, aiming at the sustainable and cost-effective production of isoprenoids for various industrial applications.

Bacteria and yeast systems provide controlled and scalable terpenoids production through fermentation and targeted engineering. The optimization strategies of terpenoids production by metabolic engineering are usually classified into upstream, midstream and downstream modifications. Upstream metabolic pathway strategies focus on increasing the availability of universal isomeric precursors, IPP and DMAPP, improving the catalysis rate or overexpressing MVA and MEP enzymes (mainly HMGR and acetyl-CoA synthase), increasing substrate availability, and inhibiting competing pathways. Among the strategies based on MVA pathway enzymes, increasing the supply of acetyl-CoA can be achieved through overexpressing of the acetyl-CoA synthase gene [219,220], enhancing the pentose phosphate pathway by overexpressing related enzymes [221], and downregulating fatty acids synthase [222] and sterol synthesis pathway enzymes [221].

The regulation of HMGS and HMGR, such as inducing their overexpression [223,224] and inclusion of a truncated HMGR (tHMGR) indifferent to sterol-mediated negative feedback, are also well-established strategies [225,226]. Overexpressing IDI/IDI1 and the insertion of mutants with higher catalytic activity have also enabled the optimization of terpenoid biosynthesis [220,223,227,228,229]. Finally, although less explored, the overexpression and introduction of PMK [226,230], MDD and acetoacetyl-CoA thiolase (ERG10) [229] have led to higher yields of terpenoid production. Strategies for optimizing terpenoids production through the MEP pathway include increasing the expression and/or improving the activity of DXS and DXR [231,232,233].

Midstream metabolic pathway optimization strategies focus on improving the catalytic efficiency and specificity of terpene synthases. Targeted mutagenesis of the enzyme’s active site is a valid strategy to increase biosynthesis and optimize the yield of the desired products [234,235,236,237,238,239,240,241], as well as enhance catalysis [235]. Two-phase fermentation systems can facilitate product extraction and adsorption, considering the low solubility of terpenes in aqueous media, therefore improving the production of these molecules [242]. Downstream metabolic engineering mainly aims at the functional modification of terpenoids by terpenoid-modifying enzymes. These enzymes are frequently associated with low catalytic efficiency, formation of undesired products, and low structural stability. The main terpenoid-modifying enzymes, such as CYPs and glycosyltransferases (UGTs), are frequently explored in metabolic engineering strategies and promote the conversion of precursors into the desired products.

Combinations of the strategies have been successfully employed to reach suitable levels of heterologous terpenoid production for industrial applications. Notable examples of highly optimized microbial biosynthesis from the natural sweeteners, food additives, and flavoring industries include: the production of stevioside (1.14 g/L in *S. cerevisiae*) [243], nootkatone (16.6 g/L in *S. cerevisiae*) [244], lycopene (3.28 g/L in *S. cerevisiae* and 4.2 g/L in *Yarrowia lipolytica*) [230,245], limonene (2.23 g/L in *S. cerevisiae* and 7.3 g/L in *E. coli*) [208,246] and linalool (4.16 g/L in *E. coli*) [247]. Optimized production of nutraceutical, aquaculture, and cosmetic terpenoids include astaxanthin (0.86 g/L in *Yarrowia lipolytica*) [245], squalene (35 g/L in *Yarrowia lipolytica*) [248] and geraniol (1.1 g/L in *C. glycerinogenes*) [249]. Pharmaceutical terpenoids high-yield production has also been achieved for taxadiene (1.25 g/L in *E. coli*) [250], ginsenoside (5 g/L in *S. cerevisiae*) [251] and artemisinin acid (25 g/L in *S. cerevisiae*) [252]. High-yield production of industrial chemicals and specialty materials terpenoids, such as β-farnesene (130 g/L in *S. cerevisiae*) [210] and sclareol (12.9 g/L in *Yarrowia lipolytica*) [253] have also been successfully optimized. It is worth highlighting the following excellent reviews recently published on terpenoid biosynthesis optimization strategies [98,109,134,240,254,255,256,257].

### 6.1. Escherichia Coli as the Bacteria Model Organism for the Engineering Mevalonate Pathway

The MVA pathway plays a crucial role in the biosynthesis of isoprenoids, a diverse class of natural compounds with important industrial applications [138]. Microbial production of these compounds through metabolic engineering, particularly in *E. coli*, has attracted increasing attention due to its potential for high growth rates and the availability of well-established genetic tools [138,258]. The heterologous expression of the MVA pathway enzymes in *E. coli*, a host that naturally synthesizes isoprenoids via the MEP pathway, represents a promising strategy to enhance the biosynthesis of crucial isoprenoid precursors, such as IPP and DMAPP. Effective implementation of this pathway requires careful analysis of pathway integration, regulation of gene expression, and optimization of metabolic flux [259].

The metabolic engineering of the MVA pathway in *E. coli* presents several challenges, including increased metabolic burden and the potential accumulation of toxic intermediates [259]. Advanced metabolic engineering strategies, such as fine-tuning gene expression, redirecting metabolic flux, and integrating hybrid biosynthetic routes, are essential to improving pathway efficiency and boosting production yields [138]. A broad and detailed understanding of regulatory mechanisms and metabolic interactions in *E. coli*, together with the development of innovative synthetic biology tools, continues to drive significant progress in MVA pathway engineering for industrial applications [138].

The full introduction of the MVA pathway into *E. coli* is typically achieved through plasmid-based systems or chromosomal integration [258]. Plasmids offer the advantage of a high number of gene copies, resulting in elevated levels of protein expression and ease of genetic manipulation. Various plasmid backbones with different origins of replication and antibiotic resistance markers are available, facilitating the introduction and maintenance of the MVA pathway within the bacterial host [258]. In contrast, chromosomal integration provides enhanced genetic stability, making it a suitable approach for long-term or large-scale applications [258]. Precise control of MVA pathway activity is essential to optimize production and mitigate potential toxic effects. This control is achieved using different types of promoters (constitutive or inducible), modulation of ribosome binding site (RBS) strengths, and the implementation of dynamic regulatory mechanisms [260].

The expression and activity of key enzymes, particularly HMGR and MVK, are critical determinants of the flux through the MVA pathway. HMGR catalyzes a rate-limiting step in the pathway, and its structural modification through truncation of regulating domain, variant selection, and codon optimization, has been shown to enhance mevalonate production in *E. coli* [261]. MVK, which catalyzes the phosphorylation of mevalonate, can be optimized through high-level expression, directed evolution, and the selection of feedback-resistant variants [262]. The elimination or modification of competing metabolic pathways in *E. coli* is an effective strategy to increase flux through the MVA pathway [258]. By redirecting the metabolic flow of essential precursors toward the MVA pathway, isoprenoid production can be significantly enhanced. Studies have demonstrated the effectiveness of this approach through targeted gene knockouts or the use of tools like CRISPR to downregulate competing pathways [258]. Redirecting metabolic flux by eliminating or reducing the activity of competing routes remains a powerful strategy for improving the biosynthesis of MVA-derived compounds [258].

Engineering the MVA pathway in *E. coli* for isoprenoid production encompasses a wide range of strategies, including pathway introduction, regulation of gene expression, optimization of key enzymes, redirection of metabolic flux, and the integration of hybrid pathways. Despite significant progress, challenges such as metabolic burden and intermediate toxicity remain major constraints. The use of advanced synthetic biology tools and system-level approaches are essential for comprehensive pathway optimization. Additionally, the development of novel host strains with enhanced tolerance to isoprenoid intermediates is expected to play a critical role in improving production efficiency and industrial scalability.

### 6.2. Saccharomyces Cerevisiae and Komagataella Phaffii as Relevant Eukaryotic Chassis Organisms

In genetic and metabolic engineering, yeasts such as *S. cerevisiae* and *Komagataella phaffii* (formerly named *Pichia pastoris)* have emerged as widely relevant model organisms for the biosynthesis of terpenes and steroids [263]. *S. cerevisiae* is a well-characterized eukaryotic microorganism and possesses a robust set of genetic tools that allow its engineering to produce various compounds of interest [161,218]. Among its main advantages are its well-elucidated genetics, ease of manipulation, and the endogenous availability of essential metabolic precursors [210,215,217]. *K. phaffii* is widely employed in the production of recombinant proteins and has emerged as a promising platform for the synthesis of chemical and natural compounds [263]. One of its main advantages is its ability to reach high cell densities in fermentation processes, which makes it attractive for applications in industrial biotechnology [264].

One approach to modifying the MVA pathway is the overexpression of the truncated form of HMG-CoA reductase (tHMGR), a rate-limiting enzyme in the pathway, which is widely used [218]. A recent study conducted by Surui Lu et al., in 2022, used a metabolomics-guided strategy to identify new genetic targets associated with the MVA pathway in *S. cerevisiae*. Variants of HMGR from other organisms, such as the truncated MvaE from *Enterococcus faecalis* (EfHMGR), demonstrated superior performance compared to the native enzyme, promoting up to a 431-fold increase in squalene accumulation in haploid yeast and a 9-fold increase in industrial strains. Metabolic analyses also revealed a positive correlation between MVA pathway flux and β-alanine metabolism [161].

In 2025, Ge et al., engineered a *S. cerevisiae* cell factory that couples an orthogonal isopentenol-utilization pathway (IUP) with compartmentalized precursor synthesis and an artificial multifunctional enzyme to produce the diterpene precursor miltiradiene. Targeting the IUP to mitochondria, peroxisomes, and the cytoplasm increased the GGPP pool 1.5-fold and, when combined with peroxisomal MVA pathway expression, raised miltiradiene titers to 146.1 mg/L (11-fold over the cytoplasmic baseline) [265]. Fusion of the prenyltransferase domain PvPT to tSmCPS-tSmKSL yielded a 2.8-fold boost to 414.4 mg/L and fed-batch fermentation in a 5 L bioreactor achieved 1.02 g/L (60.8-fold increase) [265]. The study demonstrates that the combined strategy of orthogonal pathway placement and enzyme assembly line catalysis provides a robust chassis for high-value diterpene production in yeast and can be extended to other plant-derived compounds.

In this context, the most sought-after compounds are the scarce ones, such as β-Caryophyllene. It is a bicyclic sesquiterpene with anti-inflammatory, anticancer, analgesic, and high-energy-density biofuel properties, scarce in plants, which makes microbial synthesis attractive. In 2025, Cheng et al., engineered *K. phaffii* to produce β-caryophyllene *de novo*. Codon-optimized AaCPS was integrated into the genome, yielding 3.6 mg/L [266]. Fusion of ERG20 to AaCPS with a (PA)_5_ linker doubled production to 8.4 mg/L. “Push-pull” enhancement of the mevalonate pathway (tHMG1, IDI1, ERG12, ERG19) raised titers to 32.3 mg/L, while truncating the ERG9 promoter (100 bp) further increased output to 44.3 mg/L [266]. Finally, multi-copy integration of ERG20-(PA)_5_-AaCPS produced 136.4 mg/L, a 37-fold rise over the initial strain. The engineered *K. phaffii* strain exhibited the highest reported β-caryophyllene titers in this host and validated a modular engineering pipeline for a scalable platform for sesquiterpene production, in addition to constituting a blueprint for other high-value terpenoids.

Another important aspect that directly influences the increase in metabolic flux is the availability of pathway precursors, with acetyl-CoA being a direct precursor of the MVA pathway [161]. In *K. phaffii*, a study aimed at increasing the intracellular supply of acetyl-CoA utilized the PK pathway along with a heterologous xylose utilization pathway or the endogenous methanol utilization pathway, to produce triacetic acid lactone (TAL) [267]. TAL is a cyclic structure derived from triacetic acid that represents a sustainable polyketide alternative with high potential for biotechnological applications. The integration of the polyketide pathway with xylose metabolism resulted in the production of 825.6 mg/L of TAL in minimal medium containing only xylose as the carbon source, achieving a yield of 0.041 g of TAL per gram of xylose consumed [267].

However, these pathway optimization and modification approaches may encounter obstacles depending on the metabolic pathway chosen, as demonstrated in the study conducted by Xiaojing Jiang and collaborators (2024). By using CRISPR to overexpress HMG1, it was shown to be part of a rate-limiting step in geraniol production in *K. phaffii* [268]. Recent advances in mevalonate pathway engineering in *S. cerevisiae* and *K. phaffii* demonstrate the enormous potential of these yeasts as cell factories to produce high-value isoprenoids. Despite significant progress, challenges remain to be overcome and future directions to be explored.

### 6.3. Engineering Non-Model Microorganisms for Mevalonate and Other Terpenoids Production

Although the focus has been on engineering model organisms like *E. coli* and *S. cerevisiae*, non-model microorganisms are increasingly being recognized for their unique features [205]. Their advantages often include metabolic pathways to use a wider range of carbon sources, tolerance to extreme environmental conditions, and novel enzymes and regulatory mechanisms that can enhance mevalonate production.

The bacterial domain presents a diverse background of mevalonate production, with handful non-model organisms demonstrating significant potential through metabolic engineering. An advantage is to explore alternative substrates used for mevalonate synthesis in engineered strains. This is the case with *Pseudomonas putida*. Recent works have explored the use of alternative substrates such as ethanol [269] and 2,3-butanediol [270] as the carbon source. Furthermore, the *P. putida* KT2440 strain has been engineered to improve mevalonate yields on glycerol and xylose substrates [271]. As an approach to sustainable mevalonate production, *Ralstonia eutropha* (*Cupriavidus necato*) is a facultative chemolithoautotrophic bacterium capable of using CO_2_ and H_2_ as its sole carbon source [272]. Researchers developed a plasmid system enabling the production of approximately 10 g/L of mevalonate directly from CO_2_ [137].

Extremophilic bacteria also composes an interesting group for next generation industrial biotechnology. One of its members, *Halomonas bluephagenesis*, is a halophilic and salt-tolerant bacteria with dominant growth at unsterile open fermentation conditions [273]. This organism integrating exogenous MVA synthesis genes was designed, optimizing metabolic fluxes with a mixed carbon source (glucose and acetic acid) and down-regulating competing pathways with CRISPR interference [274]. Additionally, another gene expression system based on small regulatory RNAs was developed to increase mevalonate production [275] and to introduce a heterologous MVA pathway for lycopene production [276].

The oleaginous yeast *Yarrowia lipolytica* is a promising host for terpenoid production [277,278]. This yeast associated advantages include: a sequenced genome, capacity to grow in several substrates, GRAS-status, and synthetic biology tools [279]. Notably, what makes this microorganism particularly promising is its hydrophobic microenvironment [280]. Therefore, researchers have been applying a broad range of strategies to enhance terpenoid production. Among them are the direct alteration in the MVA pathway with gene overexpression, such as HMG-CoA, ERG1/9, and IDI [281,282,283]. Indirect improvements include the modulation of lipid storage [230,284] and pathway compartmentalization [285].

Notably, other non-modal fungal species are also being studied and are worth highlighting. The red yeast is a group of interest due to its ability to produce microbial lipids and carotenoids. Among them are *Xanthophyllomyces dendrorhous* [286], *Rhodosporidium toruloides* [287,288], and *Rhodotorula mucilaginosa* [289]. Finally, the anaerobic fungus *Piromyces indianae* presents homologous enzymes such as PI.atoB that have been expressed in *E. coli* [290].

Photosynthetic organisms, such as Microalgae and Cyanobacteria, present other types of microorganisms aimed at mevalonate production due to its metabolic production derived from CO_2_ and light [291,292]. In microalgae, most works rely on *Chlamydomonas reinhardtii* and *Phaeodactylum tricornutum* [293,294]. Thus, using *C. reinhardtii*, a complete MVA pathway [295], an isopentenol utilization pathway [296], and plant isoprene synthases were introduced [297]. *P. tricornutum* has been used to express plant enzymes [298] and overexpression of endogenous genes [299,300,301] to terpenoids production. Other relevant algae that have been engineered for isoprenoid production include *Chlorella zofingiensis* [302], *Dunaliella salina* [303,304], *Fistulifera solaris* [305], *Haematococcus pluvialis* [306,307], *Nannochloropsis oceanica* [308], and *Scenedesmus* sp. CPC2 [309].

In Cyanobacteria, research to enhance terpenoid production has focused mainly on a handful of species, such as *Synechocystis* sp. PCC 6803 and *Synechococcus elongatus* (PCC 7942 and UTEX 2973) [310,311]. Using *Synechocystis* sp., the strategies include overexpression of endogenous enzymes of the MEP pathway [312], heterologous expression along with an isoprene synthase (IspS) fused to the cpcB protein [313], and the use of computational strain design techniques with metabolic flux analysis [314]. Based on *S. elongatus*, the MEP pathway was engineered by introducing plant isoprene synthases followed by upregulation of endogenous genes [315].

Despite significant advances in the production of mevalonate and other terpenoids in non-model organisms, much remains to be done. The improvement and development of synthetic biology tools, as well as a solid understanding of the genome and metalloids of these new organisms, are needed to take advantage of their unique and promising characteristics.

### 6.4. Cell-Free Systems and the Mevalonate Pathway

Cell-free systems can be often defined as platforms in which biochemical reactions occur independently of living organisms; therefore, they bypass the restrictions imposed by energy balancing flux requirements, and cell growth and viability constraints. These systems can encompass the use of purified enzymes or crude cell lysates [316]. Recently, cell-free technologies have emerged as a promising bottom-up approach for the biosynthesis of natural products in industrial biotechnology. These cell-free technologies offer solutions to several limitations associated with extracting natural products from their native producers, including fluctuations in yield due to environmental factors, such as climate variability, the extensive area required for economically viable production, and the associated environmental impacts. Furthermore, cell-free synthetic biology can address challenges inherent to heterologous expression, such as host cytotoxicity and the difficulty of identifying genomic safe harbors capable of accommodating the multiple genes required to reconstruct complex biosynthetic pathways [317].

Regarding the mevalonate pathway, initial attempts for its in vitro reconstitution consisted of Taguchi orthogonal array design to guide the best combinatorial assembly of the pathway’s main enzymes (Erg12:Erg8:Erg19:Idi:IspA) for the synthesis of amorpha-4,11-diene (AD), a key precursor to artemisinin [318]. In the subsequent year, Zhu and colleagues employed in vitro reconstitution of the MVA pathway; however, in this case, the data were utilized to optimize biosynthetic components for *Escherichia coli*-based terpenoid farnesene production [319]. Following this study, several other works adopted the use of cell lysates.

*E. coli* cell-free lysates have been used since 1948, with their main contribution when Nirenberg and Matthaei described the first codon of the universal genetic code by using them. Nowadays, *E. coli* cell extracts are the most widely used prokaryotic cell-free systems, as they are the most robust and the best well-characterized [316]. In 2015, Toogood et al. used recombinant *E. coli* cell extracts to perform a high throughput screening aiming to optimize the enzymatic steps to obtain menthol isomers from pulegone [320]. The authors stated that this one-pot biocatalytic method allowed easier optimization of each enzymatic step. In the following year, by re-conceptualizing the build step of the Design–Build–Test cycle and advancing it to a modular approach mixing three *E. coli* cell extracts overexpressing key enzymes of the mevalonate pathway, Dudley and collaborators rapidly identify the best components of this biosynthetic pathway. Then, by combining the overexpressed genes in only one strain, and using this optimized cell-free system the mevalonate was synthesized at 17.6 g/L (119 mM) over 20 h, resulting in a volumetric productivity of 0.88 g/Lh [321]. Although cell-free lysates were used in these studies, *E. coli* genomic engineering was necessary, and some of the limitations inherent to living organisms remained. However, in the latter study the ability of crude extract native metabolism to recycle excess of NADH, a limiting key component of the mevalonate pathway, indicated a potential advantage of lysate-based cell-free metabolic engineering extracts.

In addition to NADPH, ATP and acetyl-CoA are essential components of the mevalonate pathway. In 2017, Koman et al., designed a synthetic biochemical system stable for at least five days, without addition of cofactors, composed of 27 enzymes that generates both NADPH and ATP and converted glucose into monoterpenes reaching high yields and titers for limonene (12.5 ± 0.3 g/L) and pinene (14.9 ± 0.6 g/L) [321]. The authors used CoPASI to determine the main critical contributors to monoterpene production. The results showed that the total yield was close to 100% of the proposed theoretical value, suggesting little or no carbon loss due to side reactions.

More recently, Dudley et al., (2019) expanded the initial work from glucose-mevalonate to glucose-limonene highlighting the flexibility of the use of cell-free rich enzymatic lysates [322]. In this study they used nine heterologous enzymes encompassing 20 biosynthetic steps, reaching 0.66 mM (90.2 mg/gLh) limonene over 24h, a productivity of 3.8 mg/gLh. Yet using limonene as an example of an isoprenoid product of interest, Sarnik et al., (2025) addressed the constraint of enzyme stability using thermophilic enzymes to produce this compound [323]. Their results demonstrated a 1.7-fold increase in yield with Archae thermophilic-derived enzymes compared to the mesophiles variants used by Koman et al., (2017) [321], indicating that this can be a valuable alternative to increase the robustness of cell-free systems.

In conclusion, although cell-free systems have been proposed as a viable alternative for the production of natural products due to several key advantages, including one-pot biosynthesis, simplified product purification and analysis (owing to the absence of cellular components), elimination of cellular toxicity, avoidance of genomic stability constraints, and intrinsic biocontainment, they also present certain limitations. These include restricted energy and metabolite availability, limited scalability, enzyme stability and constrained access to precursor molecules [317].

## 7. Strategies for Sustainable Microbial Mevalonate Bioproduction

Currently, most of the potential MVA-derived chemicals are produced either by chemical synthesis or extraction from plant or animal tissues [161,324]. Chemical synthesis, while capable of producing specific molecules, often relies on complex, multi-step routes that require substantial energy input, use hazardous catalysts, and generate undesirable byproducts, making it neither cost-effective nor environmentally friendly [325]. Moreover, this method cannot selectively generate the (R)- or (S)-mevalonate enantiomers, producing a racemic mixture of both at an equimolar ratio, whereas only the (R)- enantiomer is bioactive. Achieving chiral purity requires additional purification steps, which further increase costs [326]. On the other hand, extraction from plant or animal tissues is limited by low target-molecule content in raw materials and variations in biomass quality, which can negatively affect conversion efficiency [327]. In contrast, microbial production of MVA offers a compelling and sustainable alternative, presenting advantages, such as lower production costs, process scalability and reduced environmental footprint [161,325].

With the global market projected to reach USD 10 billion by 2030, the shift from traditional extraction methods toward microbial cell factories is poised to play a pivotal role in advancing the bioeconomy [328]. For these reasons, industrial production of MVA and its derivatives is progressively shifting away from the traditional chemical and extraction routes toward microbial fermentation. The economic viability of processes based on the mevalonate pathway depends on the ability to engineer microorganisms (such as *Y. lipolytica* and *E. coli*) to achieve high titers and yields [166,329].

Nevertheless, significant milestones must still be reached to achieve an economically viable MVA production process based on microbial fermentation. Commercial feasibility for large-scale bioproduction is primarily determined by titer, productivity, and yield, with industrial benchmarks requiring MVA titers between 150 and 200 g/L and volumetric productivities of at least 2–3 g/Lh, for first or second-generation feedstocks [137,330]. Although many organisms have been engineered for increased MVA production, these parameters have not yet reached the necessary levels for economic viability. The highest reported production achieved 121 g/L of MVA at 120 h from acetic acid and glucose in fed-batch fermentation using a *Halomonas bluephagenesis* strain, while the highest volumetric productivity reached 1.2 g/Lh from glucose in a nitrogen-limited fed-batch fermentation using an *E. coli* strain [274,331].

In this scenario, it is important to consider possible strategies to increase production and reduce costs in MVA production processes. Chassis selection, metabolic engineering and process optimization represent the key approaches toward these goals. Regarding chassis selection, yeast and bacteria have been the primary microorganisms used for MVA production, though alternative hosts such as algae have recently been engineered for autotrophic MVA synthesis [295,326]. Yeasts ubiquitously possess a native MVA pathway, which diverts MVA to other cellular processes; whereas, the most widely used bacteria for bioproduction, such as *E. coli* and *P. putida*, lack this pathway. Archaea have previously been used to produce isoprene from wastewater, although model strains have greater potential for MVA production due to available genomic information and gene-editing tools [135,332].

Therefore, chassis selection emerges as the primary strategic decision in industrial-scale MVA production, as it determines the availability of precursor pools, compatibility with existing metabolic flux, and the extent of engineering required to achieve commercially relevant titers. Nevertheless, metabolic engineering remains an indispensable tool for achieving higher bioproduct titers. In this field, efforts to increase MVA production are mainly focused on transcriptional tuning of endogenous MVA pathways, the bioprospection of highly active genes for heterologous expression, enhancing the availability of the cofactors acetyl-CoA and NADPH, and minimizing MVA flux toward competing native pathways [135,326]. Other techniques, such as enzyme and ribosome-binding site engineering, have also been used successfully to improve MVA titers [333,334].

However, despite the array of genetic manipulation tools available to enhance MVA production, these efforts have proven insufficient for the development of industrial-scale processes. Scaling up processes with these engineered strains remains a challenge, as microorganisms in industrial settings face environmental stresses that are not considered at the laboratory scale, leading to reduced titers, yield and productivity [335]. In this scenario, process and media optimization are also effective strategies to reduce the overall value of the production process.

In the realm of process optimization, different operation modes and nutrient modulation are among the most employed strategies to increase MVA production [326]. Considering fermentation modes, batch fermentation occurs in a closed system in which no additional feedstock is added beyond that provided at the start of the process. This allows for simple operation and reduces the risk of contamination, as there is no entry or removal of nutrients [336]. However, while simple, batch mode often results in lower titers due to substrate depletion and the accumulation of inhibitory by products, which include both extracellular overflow metabolites and intracellular pathway intermediates [166,295,326]. The intermediate HMG-CoA, for example, is highly cytotoxic at elevated levels and has been shown to inhibit fatty acid biosynthesis, membrane formation, and overall cell viability [329,337]. In addition, isopentenyl pyrophosphate IPP and DMAPP also negatively affect cell growth and viability and cause plasmid instability in bacterial hosts [325,338]. In eukaryotic hosts, mevalonate 5-phosphate (MVAP) and mevalonate 5-pyrophosphate (MVAPP) are specifically toxic to mitochondria [325]. These pyrophosphate compounds can react with amino acids to form toxic ATP analogs that inhibit essential mitochondrial enzymes [324].

In contrast, fed-batch operation employs a feeding strategy in which substrates are gradually supplied to the bioreactor during cultivation. This fermentation process allows the maintenance of optimal substrate concentrations for final product synthesis, thereby improving production metrics [336]. As the industrial standard for high-level production, this mode has enabled record-breaking results, with titers of MVA above 100 g/L in both bacteria and yeast [166,274,339]. Other fermentation modes, such as two-stage fermentation, have also been employed to increase MVA production. In two-stage fermentation, growth and production are decoupled, minimizing metabolic burden and toxicity [326]. In *E. coli*, for example, when acetate was used as a carbon source for MVA production in a fed-batch strategy, this substrate inhibited bacterial growth. On the other hand, when a two-stage fermentation was employed, with glucose as the feedstock for biomass accumulation in the first stage and acetate as the sole carbon source in a subsequent production phase, MVA titers increased more than 7-fold [340].

Then, optimization of the cultivation environment is necessary to reduce energy consumption and improve yields [341]. While submerged fermentation is the industrial standard due to precise control over pH, temperature, and oxygen, solid-state fermentation is recognized for its lower energy requirements and reduced wastewater generation, especially for secondary metabolites like statins [170,341] using agro-industrial residues with lower energy consumption and less wastewater generation [170,342]. However, it faces challenges regarding heat and mass transfer at a large scale [343].

In addition to operating modes, critical fermentation parameters have a deep impact on titer, yield and productivity [336], as ensuring optimal conditions for growth and MVA production is essential for achieving higher output. Aeration rate, for example, is critical for balancing cell growth and MVA production, as higher oxygen concentrations allow the diversion of acetyl-CoA to the citric acid cycle, reducing its availability for MVA synthesis. In contrast, lower oxygen availability directs more acetyl-CoA to MVA production, resulting in higher yields [270,326]. At excessively low aeration rates, however, there is insufficient oxygen for cell growth and maintenance metabolism, thereby decreasing MVA production [326]. Therefore, dissolved oxygen levels must be optimized to maintain biomass formation while minimizing acetyl-CoA diversion.

Substrate concentration also significantly influences the balance between MVA titers, yields, and overall cell fitness. Increasing the concentration of a primary substrate, such as glucose, typically leads to higher product titers but often results in reduced yield. For example, in *S. cerevisiae*, increasing glucose from 2% to 6% resulted in a 1.7-fold increase in mevalonate titers, but the yield dropped from 31.2% to 17.3% of the theoretical maximum [344]. This reduction in yield at high concentrations is largely attributed to overflow metabolism, in which carbon flux is diverted into side products such as ethanol and glycerol rather than the mevalonate pathway [326,344]. Additionally, pH, temperature and inoculating volumes must be adjusted for all fermentations [135,336].

In addition to process optimization, reducing feedstock costs is critical to ensure the economic viability of industrial bioprocesses. In large-scale fermentation, the use of pure carbon sources such as glucose makes the process highly costly; hence, current efforts lean toward the use of more abundant and inedible substrates [335,336]. While glucose remains the most common carbon source for mevalonate production, researchers have explored inexpensive or waste-derived materials such as glycerol, lignocellulosic residues, acetate, methanol and CO_2_ [135,137,326].

Glycerol is an abundant and low-cost byproduct of the biodiesel industry, whose production has grown rapidly in recent years, with annual biodiesel production exceeding 68 million metric tons in 2024 [345]. The transesterification process used in biodiesel production yields around 10% (w/w) crude glycerol, and improper disposal of this residue can lead to environmental pollution, contaminating soil and water bodies [345]. In this context, glycerol represents a renewable and widely available feedstock for the microbial production of high-value compounds such as MVA [346]. However, its use in MVA production is constrained by the loss of one carbon atom during acetyl-CoA synthesis, thereby limiting overall yields [326]. Despite this limitation, its use as a carbon source for MVA production has been successfully demonstrated in both *E. coli* and *P. putida*, highlighting its potential as a sustainable platform for value-added biosynthesis [337,346,347].

Lignocellulose, which can be obtained from agro-industrial residues, is a low-cost, readily available alternative feedstock for MVA production [348]. Due to its recalcitrant nature, lignocellulosic biomass requires pretreatment and hydrolysis to make sugar monomers readily available to microorganisms in bio-based processes [335]. Corn stover and peanut hulls hydrolysates have been previously used as feedstocks for MVA production in *R. toruloides* and *E. coli*, respectively [348,349,350]. Cellulose-derived cellobiose has also been used as a carbon source for isoprene production in *C. cellulolyticum* and *E. coli* [351,352]. These studies focus on utilizing the sugars available in lignocellulosic structure, but aromatic lignin-derived compounds have also been used to produce MVA by *P. putida* and *Acinetobacter baylyi* [353,354].

Besides those carbon sources, other low-cost and sustainable feedstocks have been explored for MVA production. Methane is a highly polluting compound with a prominent role in climate change, and efforts have been made to convert it to MVA using engineered methanotrophs such as *Methylococcus capsulatus* [355]. Similarly, carbon dioxide, another greenhouse gas that contributes to global warming, can be directed toward MVA production, thereby reducing its presence in the atmosphere [137]. To this end, photosynthetic cyanobacteria of the genus *Synechocystis*, chemolithoautotrophic bacterium *Cupriavidus necator* and alga *Chlamydomonas reinhardtii* have been engineered to express the MVA pathway using CO_2_ as a feedstock [137,356]. Oleaginous hosts like *Y. lipolytica* can utilize waste oils or specific fatty acids such as oleic acid to produce MVA-derived β-farnesene, avoiding carbon loss in the form of CO_2_ that occurs when using glucose [204]. Acetate is another inexpensive feedstock that offers the advantage of direct conversion to acetyl-CoA without carbon loss [326]. Different bacteria, including *E. coli* and *P. putida*, have been engineered to produce MVA from acetate [135,274,326,357]. Finally, even wastewater has been used as a substrate, as demonstrated by the production of isoprene in *Methanosarcina acetivorans*, a methanogenic archaeon [332].

Therefore, to ensure the economic viability of industrial MVA production, the transition from purified feedstocks like glucose to more abundant, low-cost, and sustainable alternatives is urgent. While each substrate presents unique advantages and drawbacks, process feasibility using alternative carbon sources has been validated for various microbial hosts, paving the way for scalable and sustainable bioproduction of MVA and other high-value isoprenoids. Nevertheless, while the selection of low-cost feedstocks is essential for reducing upstream expenses in bioprocesses, downstream processing, encompassing product recovery, purification, and formulation, often represents the majority of total production costs [335]. In this context, enhancing the competitiveness of MVA production processes also depends on reducing downstream processing costs.

Industrial scaling faces significant biological and technical bottlenecks. Increasing the flux in the MVA pathway can lead to the accumulation of precursors such as isopentenyl diphosphate and dimethylallyl diphosphate, which are cytotoxic and inhibit cell growth [358,359]. Strategies involving dynamic regulation and compartment engineering (such as utilizing mitochondria) are used to mitigate these effects [359,360]. In vitro enzymatic processes are being explored to bypass cell growth inhibition entirely, allowing for rational optimization of multi-enzyme pathways without the constraints of cellular nutrient limitations or metabolic by-products [361]. For volatile products (e.g., isoprene) or hydrophobic products, simultaneous separation techniques during cultivation are crucial. The use of organic phases (such as nonane) or polymeric adsorbents (such as Diaion HP20) allows for continuous product recovery, reducing toxicity in the culture medium and increasing final yields [362]. Also, many MVA-derived products are insoluble and lighter than water, naturally forming a top oil phase in the fermenter. These characteristics can be exploited to significantly lower downstream separation costs compared to energy-intensive chemical methods [242].

For industrial applications, mevalonolactone (MVAL) is preferred over its free acid form (MVA), yet direct microbial production of MVAL is not available [326]. Instead, MVAL is produced through chemical conversion, as MVA undergoes spontaneous lactonization under acidic conditions. Thus, following fermentation, the recovered MVA undergoes a low-pH, heat-induced cyclization step to obtain MVAL [326]. Sulfuric acid has traditionally been used as a catalyst for this reaction, but, in addition to MVAL, it generates anhydro-MVAL, an undesirable byproduct that reduces downstream processing efficiency [326]. Hence, phosphoric acid was tested as an alternative acid catalyst for the conversion of MVA to MVAL. The reaction with phosphoric acid not only presented higher MVAL yields than sulfuric acid at the same ratio, but also did not produce any anhydro-MVAL, making it suitable as an industrial catalyst in this reaction [363]. In the same study, before acid conversion to MVAL, the fermentation broth was decolorized to remove contaminants and obtain high-purity MVA [363]. For this step, activated powder charcoal of different mesh sizes were evaluated, and both 20–60 and 100 mesh types resulted in completely decolorized broth. After decolorization and acid lactonization, (R)-MVAL, the bioactive enantiomer, was obtained with a recovery over 98% and 99% purity, establishing the basis for an industrial-scale downstream process for MVA [363].

High hydrophobicity and volatility generally make isoprenoids difficult to recover after fermentation [364]. It is essential, nevertheless, to prevent product loss due to evaporation to achieve an economically viable process. In situ product recovery has been extensively applied in the production of isoprenoids, a method in which products are removed from the fermentation broth during ongoing production [364]. Overlaying organic solvents in a two-phase fermentation, for example, efficiently traps hydrophobic products, such as isoprenoids, and improves final titers and yield [365,366]. A solid adsorber with high affinity for hydrophobic molecules, such as resins and other porous polymers, can also be used in a solid–liquid partitioning cultivation, which similarly captures isoprenoids efficiently, leading to higher recovery [367]. These adsorbents can be reused and are simply separated from the aqueous fermentation phase, offering advantages over the use of organic solvents for product separation, especially in an industrial setting [364]. Therefore, in situ product recovery can be explored for bio-based MVA and isoprenoids purification, as it reduces recovery stages, consequently minimizing downstream costs [364].

Therefore, the successful industrialization of MVA production will require the integration of four key pillars: (i) continued strain development through metabolic engineering and synthetic biology to enhance carbon flux, cofactor availability, and product tolerance; (ii) efficient use of alternative and cheap feedstocks; (iii) refinement of fermentation processes to optimize strain performance at scale; and (iv) development of downstream operations that minimize product loss and purification steps. By addressing these interconnected elements, microbial MVA production can transition from a promising alternative to an economically competitive and environmentally sustainable industrial reality, ultimately meeting the growing global demand for isoprenoid-based products.

## Data Availability

Data is contained within the article.

## Abbreviations

The following abbreviations are used in this manuscript:

MVA	mevalonate
MEP	2-C-methyl-D-erythritol 4-phosphate
IP	isopentenyl phosphate
IPP	isopentenyl diphosphate
DMAPP	dimethylallyl diphosphate
CoA-SH	reduced coenzyme A
acetyl-CoA	acetyl-coenzyme A
acetoacetyl-CoA	acetoacetyl-coenzyme A
TAL	triacetic acid lactone
MVAL	mevalonolactone
HMG-CoA	3-hydroxy-3-methylglutaryl-coenzyme A
MVAP	mevalonate-5-phosphate
MVAPP	mevalonate-5-diphosphate
MVABP	mevalonate-3,5-bisphosphate
G3P	D-glyceraldehyde-3-phosphate
GPP	geranyl pyrophosphate
FPP	farnesyl pyrophosphate
GGPP	geranylgeranyl pyrophosphate
AACT	acetyl-CoA acetyltransferase
HMGS	HMG-CoA synthase
HMGR	3-hydroxy-3-methylglutaryl-CoA reductase
MK	mevalonate kinase
PMK	phosphomevalonate kinase
MDD	mevalonate diphosphate decarboxylase
IDI1	type I isopentenyl diphosphate isomerase
SS	squalene synthase
SSD	sterol-sensing domain
SRE	sterol regulatory elements
SREBPs	sterol regulatory element-binding proteins
ER	endoplasmic reticulum
HRD	HMG-CoA reductase degradation pathway
GHMP	kinase superfamily of Galactokinase, Homoserine kinase, Mevalonate kinase, and Phosphomevalonate kinase
HGT	horizontal gene transfer
PDB	Protein Data Bank
LUCA	last universal common ancestor
LDL	low-density lipoprotein
HIDS	hyper-IgD syndrome
DSAP	disseminated superficial actinic porokeratosis
GRAS	microorganism Generally Recognized As Safe
IUP	isopentenol-utilization pathway
